# Supplementary material for: Quality Evaluation of Polygonatum cyrtonema Hua Based on UPLC-Q-Exactive Orbitrap MS and Electronic Sensory Techniques with Different Numbers of Steaming Cycles
Source: Foods. 2024 May 20;13(10):1586. doi: 10.3390/foods13101586 (PMC11120622; doi:10.3390/foods13101586)
Supplement: Supplementary file 1 [file foods-13-01586-s001.zip › supplement S2.pdf]

Detailed identification and analysis of compositions

Flavonoids: Flavonoids mainly undergo reverse-diels-alder reaction (RDA) cleavage. During the cleavage process, high-energy collisions cause flavonoids to lose their sugar groups and form flavonoid glycoside ions, which are then further cleaved. The quasi-molecular ion peak of peak 30 was *m/z* 271.0621 $[M-H]^-$, which is presumed to have the molecular formula $C_{15}H_{12}O_5$. Its secondary fragmentation ions were *m/z* 151.0040 $[M-H-C_8H_8O]^-$ and *m/z* 119.0501 $[M-H-C_7H_4O_4]^-$, respectively, the 2-part fragmentation ions were generated by RDA cleavage occurring in naringenin. *m/z* 107.0137 $[M-H-C_8H_8O-CO_2]^-$, is the fragment ion formed by side chain deoxygenation and C=O bonding at *m/z* 151.0040, which is presumed to be naringenin by combining the mass spectrometry cleavage pattern and literature reports, and the detailed cleavage pathway is illustrated in Figure 1 (a). The retention time of peak 25 was 3.471 min, and the quasimolecular ion was *m/z* 287.055 $[M+H]^+$, which was deduced to be $C_{15}H_{10}O_6$, and the fragmentation ion was detected at *m/z* 153.018, which was the fragment ion produced by the cleavage of kaempferol by RDA. It was determined that compound 26 was kaempferol, and the cleavage pattern is shown in Figure 1 (b).

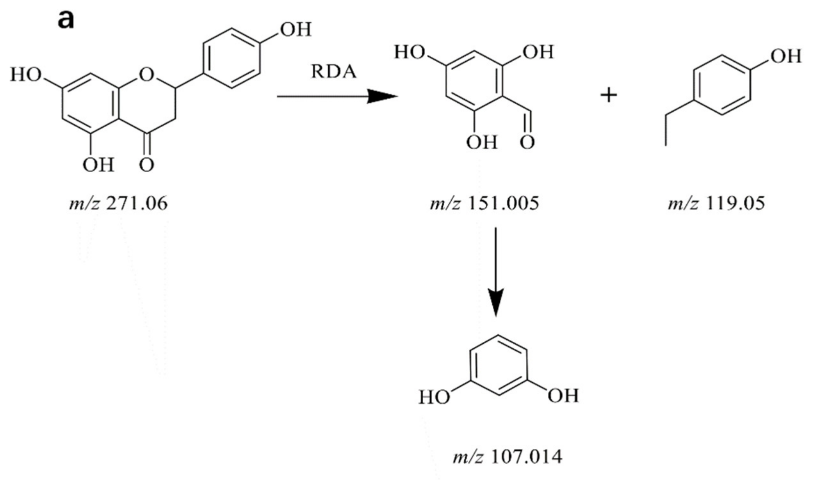

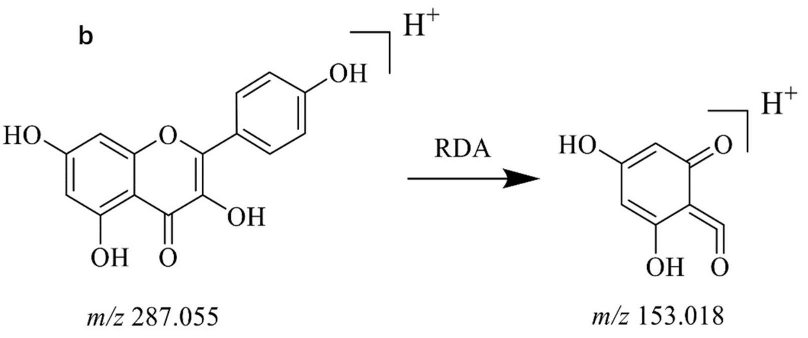

Figure S1. (a): Fragmentation pathways of naringenin; (b): Fragmentation pathways of kaempferol

Organic Acids: These compounds in the secondary mass spectrometry, the main manifestation of the loss of water molecules and carboxyl groups, or the cleavage of carbon-oxygen single bonds. 3 peak compound retention time of 0.551min, the first level of mass spectrometry revealed quasi-molecular ion peaks at *m/z* 191.020 $[M-H]^-$, suggesting a molecular formula of $C_6H_8O_7$. In the second level of mass spectrometry, a fragmentation ion at *m/z* 111.01 $[M-H-CH_4O_4]^-$ appeared, indicating the possible breakdown of the side chain carboxyl group in citric acid through the McLafferty rearrangement mechanism. Subsequently, a CO is lost to get the *m/z* 85.0298 $[M-H-CH_4O_4-CO]^-$ fragmentation ion. Based on the mass spectrometry cleavage pattern and literature reports, it is presumed to be citric acid, and the detailed cleavage pathway is illustrated in Figure 2 (a). The quasimolecular ion peak of compound 17 was *m/z* 171.02 $[M+H]^+$, indicating a presumed molecular formula of $C_7H_6O_5$. The secondary fragment ions appeared as *m/z* 153.055 $[M+H-H_2O]^+$ and *m/z* 127.03 $[M+H-CO_2]^+$, which were identified as gallic acid. The detailed cleavage pathway is illustrated in Figure 2 (b).

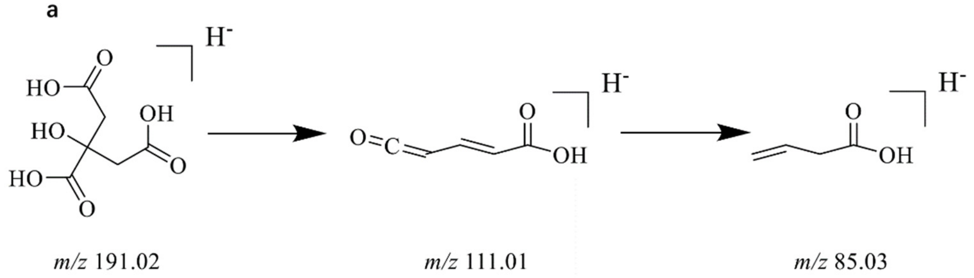

b

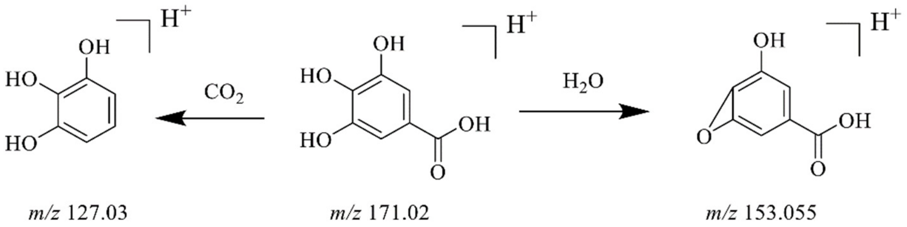

Figure S2. (a): Fragmentation pathways of citric acid; (b): Fragmentation pathways of gallic acid

Alkaloids: Alkaloidal components are primarily basic organic compounds containing nitrogen, and most of them have complex ring structures. The retention time of the peak 47 compound was 12.574 min, and the quasimolecular ion peak was *m/z* 166.086 $[M+H]^+$, which was presumed to have the structural formula $C_9H_{11}NO_2$. The fragmentation ions, *m/z* 120.08 $[M+H-CO-H_2O]^+$ and *m/z* 136.075 $[M+H-CH_2OH]^+$, appeared in the secondary mass spectrum and were lost due to the collision energies of the CO, H_2O, and CH_2OH molecules in mass spectrometry. It is presumed to be polygonatine A. The detailed cleavage pathway is shown in Figure 3 (a). The retention time of the compound at peak 54 was 0.338 min, and the quasi-molecular ion peak was *m/z* 118.086 $[M+H]^+$, which was presumed to have the molecular formula $C_5H_{11}NO_2$. It appeared in the secondary mass spectra at *m/z* 59.073 $[M+H-C_2H_3O_2]^+$ and *m/z* 58.065 $[M+H-C_2H_4O_2]^+$, presumed to be betaine. The detailed cleavage pathway is shown in Figure 3 (b).

a

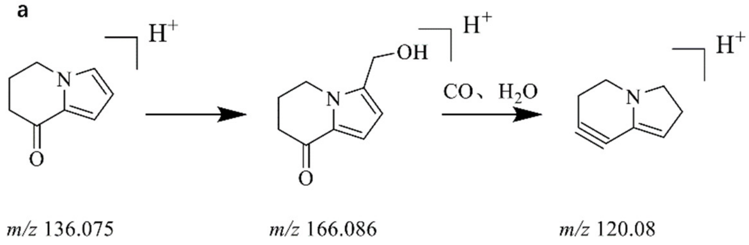

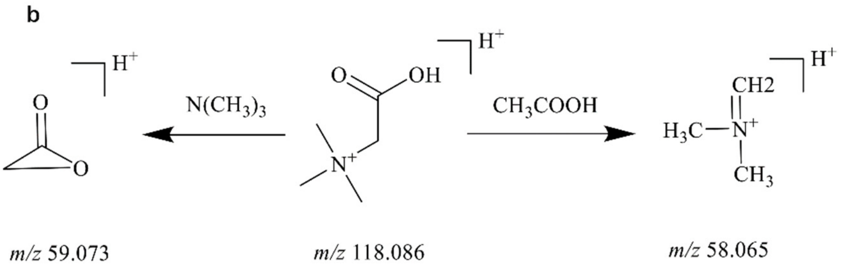

Figure S3. (a)：Fragmentation pathways of polygonatine A; (b)：Fragmentation pathways of betaine

Amino Acids: The secondary mass spectra of amino acid compounds are mainly characterized by the loss of NH_3 and HCOOH neutral ions. At 8 peak compound retention time of 0.619 min, a quasi-molecular ion peak at *m/z* 175.119 $[M+H]^+$ is presumed to represent the structural formula for $C_6H_{14}N_4O_2$. The occurrence of α-cleavage breaks in the secondary mass spectra of the fragmentation of the ions *m/z* 70.065 $[M+H-C_2H_7N_3O_2]^+$ and *m/z* 60.055 $[M+H-C_2H_7N_3O_2-CH]^+$, which is assumed to be arginine, and the detailed cleavage pathway is illustrated in Figure 4(a). The retention time of the peak 16 compound was 1.563 min, and the quasimolecular ion peak was *m/z* 164.072 $[M-H]^-$, which was presumed to have the structural formula $C_9H_{11}NO_2$. *m/z* 149.048 $[M-H-NH_3]^-$ and *m/z* 120.045 $[M-H-HCOOH]^-$ fragmentation ions appeared in the secondary mass spectrum. Based on the cleavage characteristics of the secondary mass spectrum and references, it was identified as phenylalanine. The cleavage pathway is illustrated in Figure 4 (b).

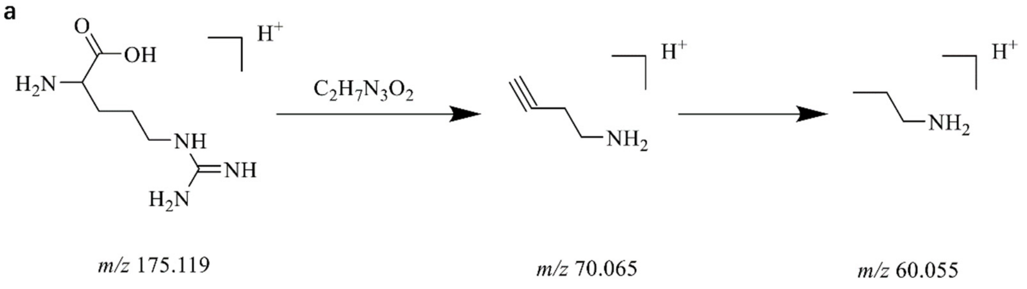

b

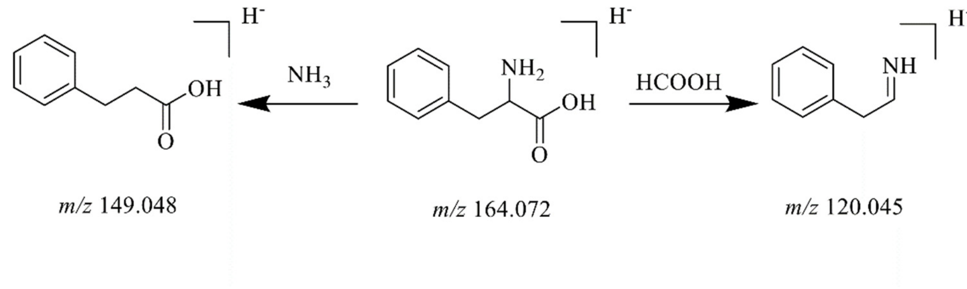

Figure S4. (a): Fragmentation pathways of arginine; (b): Fragmentation pathways of phenylalanine

Saponins: The detection of saponin compounds in the extract of PCH samples mainly focuses on steroidal saponins and triterpene saponins. At 42 peak compound retention time of 1.706 min, quasi-molecular ion peak at *m/z* 415.320 $[M+H]^+$ is presumed to represent the structural formula $C_{27}H_{42}O_{17}$. The loss of the parent ion by the EF ring results in *m/z* 271.207 $[M+H\text{-EF ring}]^+$. Subsequently, the loss of H_2O leads to *m/z* 253.195 $[M+H\text{-EF ring-}H_2O]^+$, which are characteristic fragments presumed to be diosgenin elements. The detailed cleavage pathway is illustrated in Figure 5.

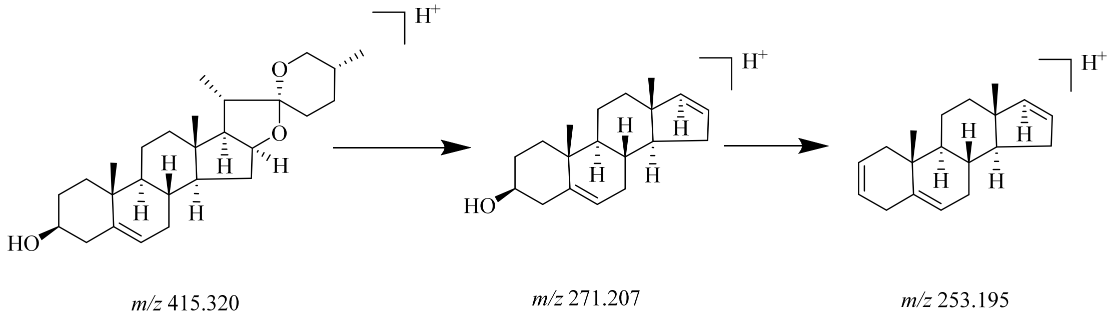

Figure S5. Fragmentation pathways of diosgenin

Other Analogues: Oligosaccharides, fatty acids, nucleosides, and other components were identified. The retention time of peak 4 compound was 0.5518 min, and the quasi-molecular ion peak was *m/z* 341.109 $[M-H]^-$, which was presumed to have the structural formula of $C_{12}H_{22}O_{11}$. The secondary mass spectrometry analysis lost one molecule of H_2O and $C_2H_8O_4$ to get *m/z* 179.056 $[M+H-C_2H_{10}O_5]^+$. It then lost a molecule of $C_3H_6O_3$ to reach *m/z* 89.025 $[M+H-C_2H_{10}O_5-C_3H_6O_3]^+$, and finally lost a molecule of CH_4O to reach *m/z* 59.014 $[M+H-C_2H_{10}O_5-C_3H_6O_3-CH_4O]^+$, which is

presumed to be sucrose. The detailed cleavage pathway is illustrated in Figure 6 (a). The fatty acid belongs to volatile oil compounds, and it easily produces COOH, $(CH_2)n$, and other fragments during cleavage. At 41 peak compound retention time was 2.985 min, and the quasi-molecular ion peak was *m/z* 281.247 $[M+H]^+$, which was presumed to represent the structural formula of $C_{18}H_{32}O_2$. The secondary mass spectra obtained at *m/z* 263.144 $[M+H-H_2O]^+$ and *m/z* 221.134 $[M+H-COOH-CH_2]^+$ were presumed to be of linoleic acid. 221.134 $[M+H-COOH-CH_2]^+$, presumed to be linoleic acid. The detailed cleavage pathway is shown in Figure 6 (b).

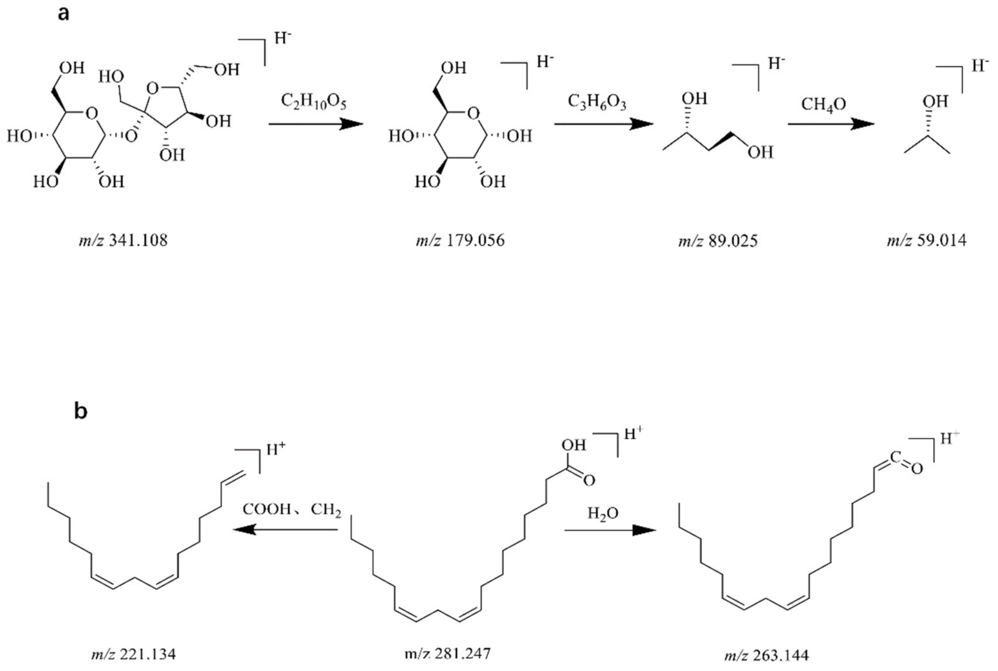

Figure S6. (a): Fragmentation pathways of sucrose; (b): Fragmentation pathways of linoleic acid
