# Supplementary figures and images for: Quality Evaluation of Polygonatum cyrtonema Hua Based on UPLC-Q-Exactive Orbitrap MS and Electronic Sensory Techniques with Different Numbers of Steaming Cycles
Source: Foods. 2024 May 20;13(10):1586. doi: 10.3390/foods13101586 (PMC11120622; doi:10.3390/foods13101586)

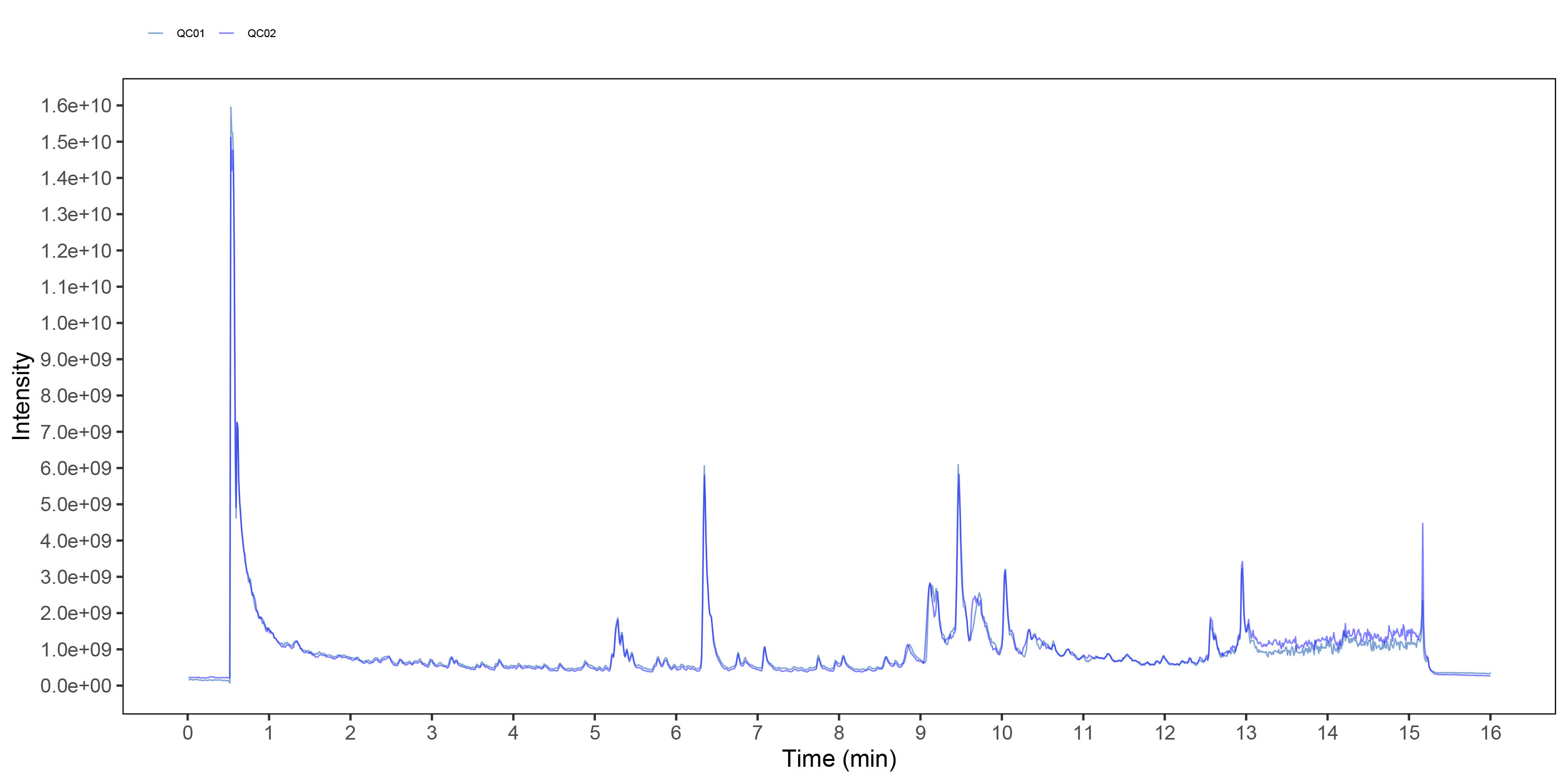

Supplement: Supplementary file 1 [file foods-13-01586-s001.zip › supplement S1/negative ion/NEG-T-1.jpg]

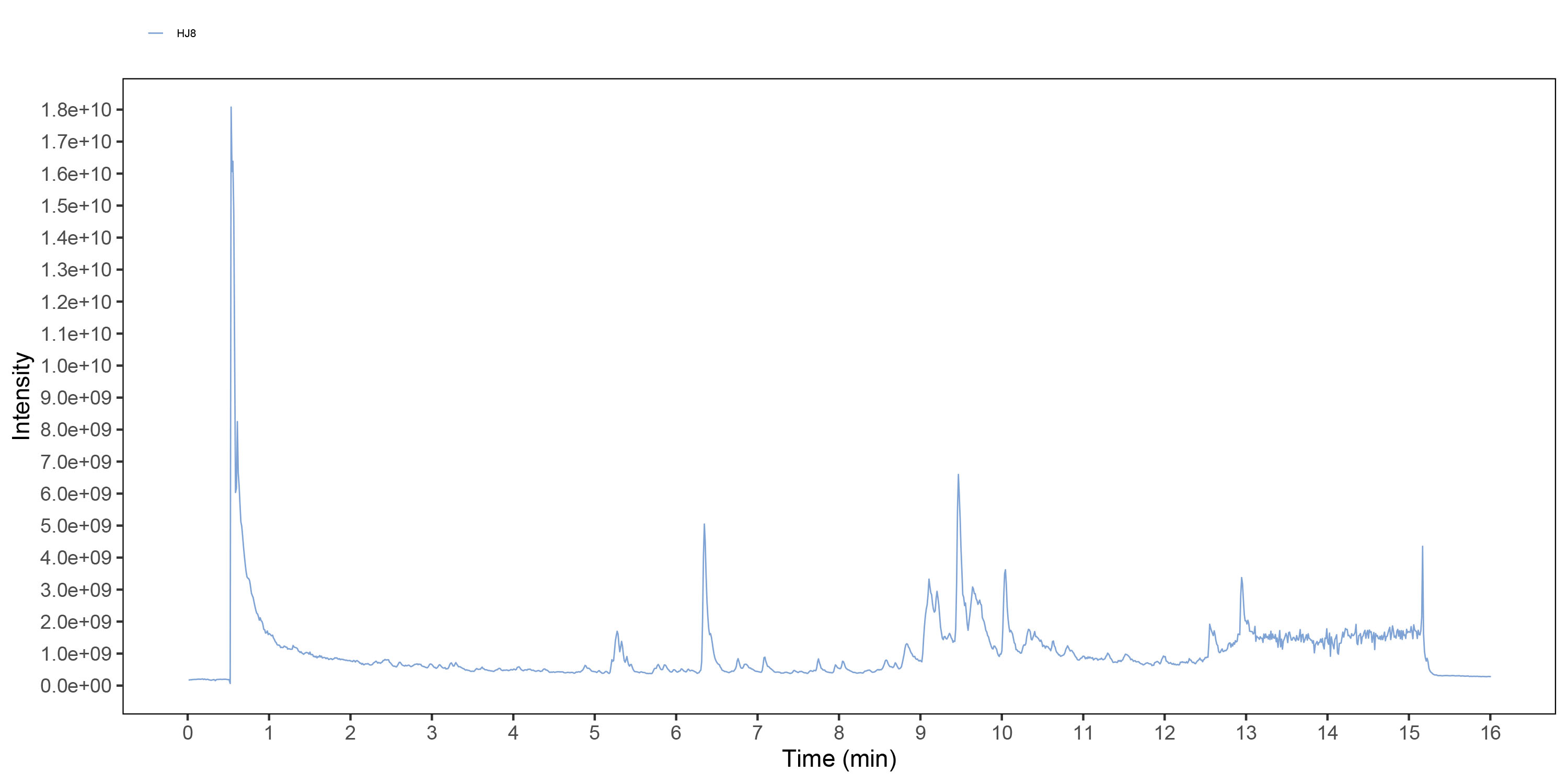

Supplement: Supplementary file 1 [file foods-13-01586-s001.zip › supplement S1/negative ion/NEG-T-10.jpg]

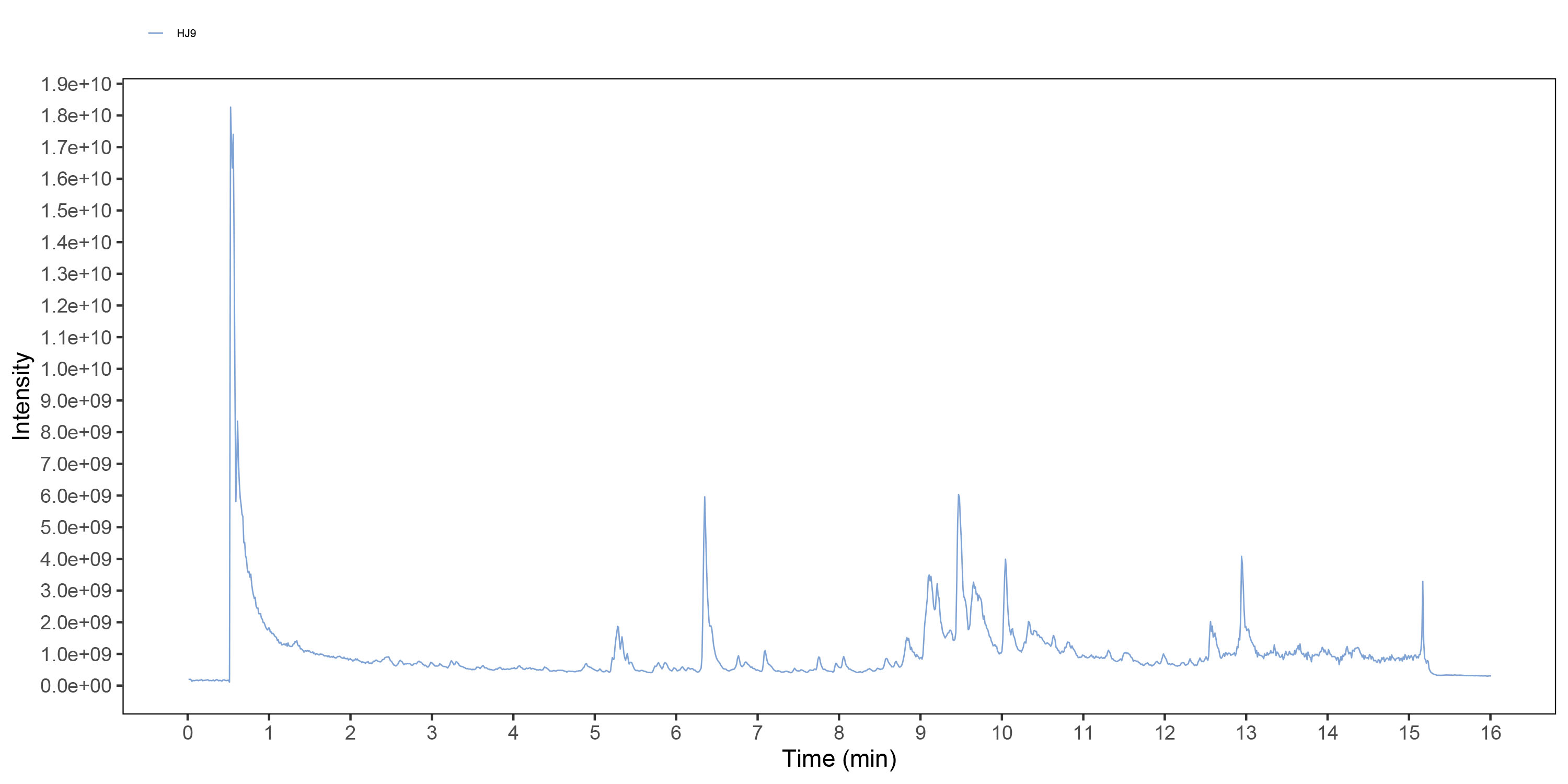

Supplement: Supplementary file 1 [file foods-13-01586-s001.zip › supplement S1/negative ion/NEG-T-11.jpg]

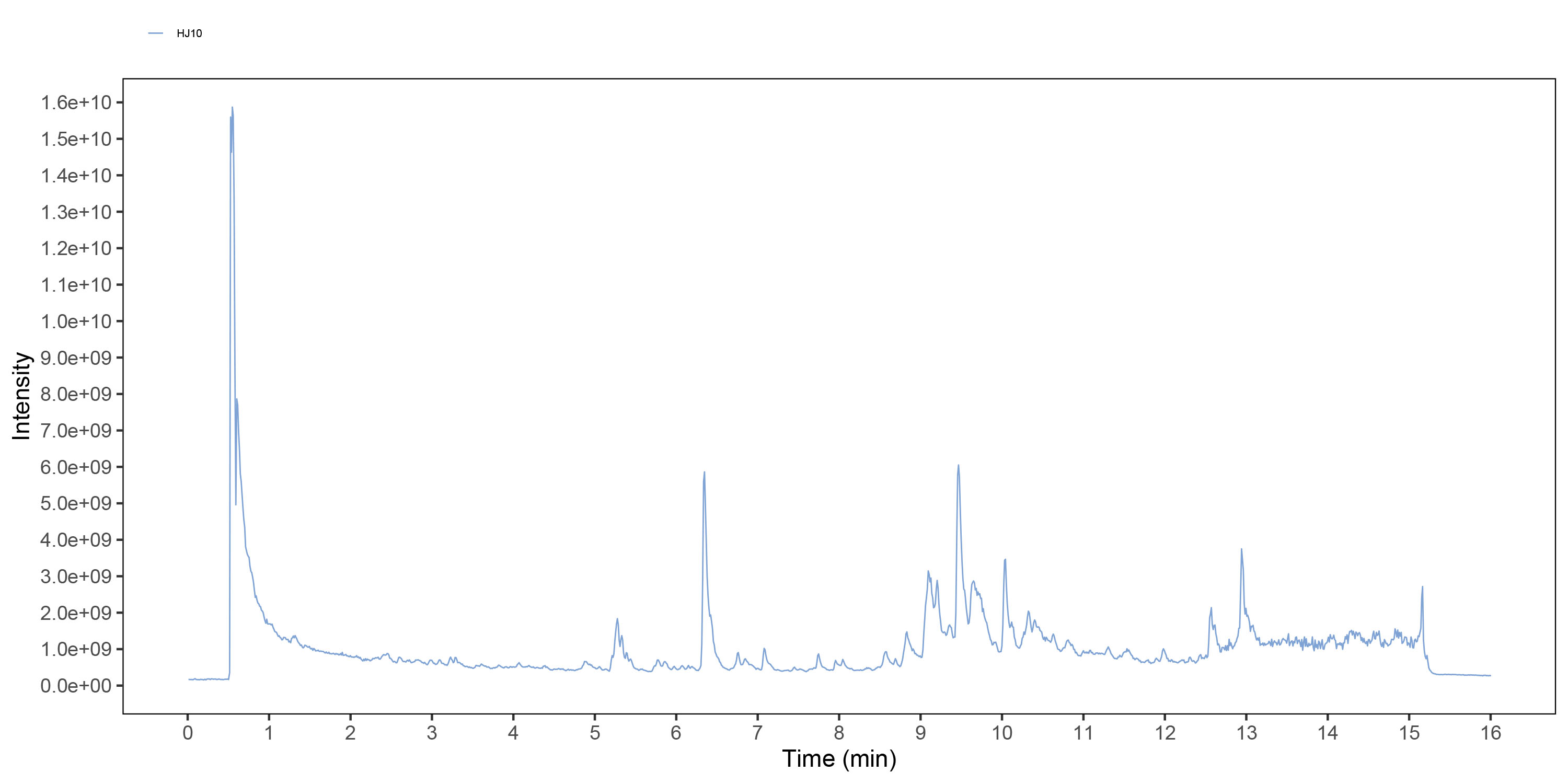

Supplement: Supplementary file 1 [file foods-13-01586-s001.zip › supplement S1/negative ion/NEG-T-12.jpg]

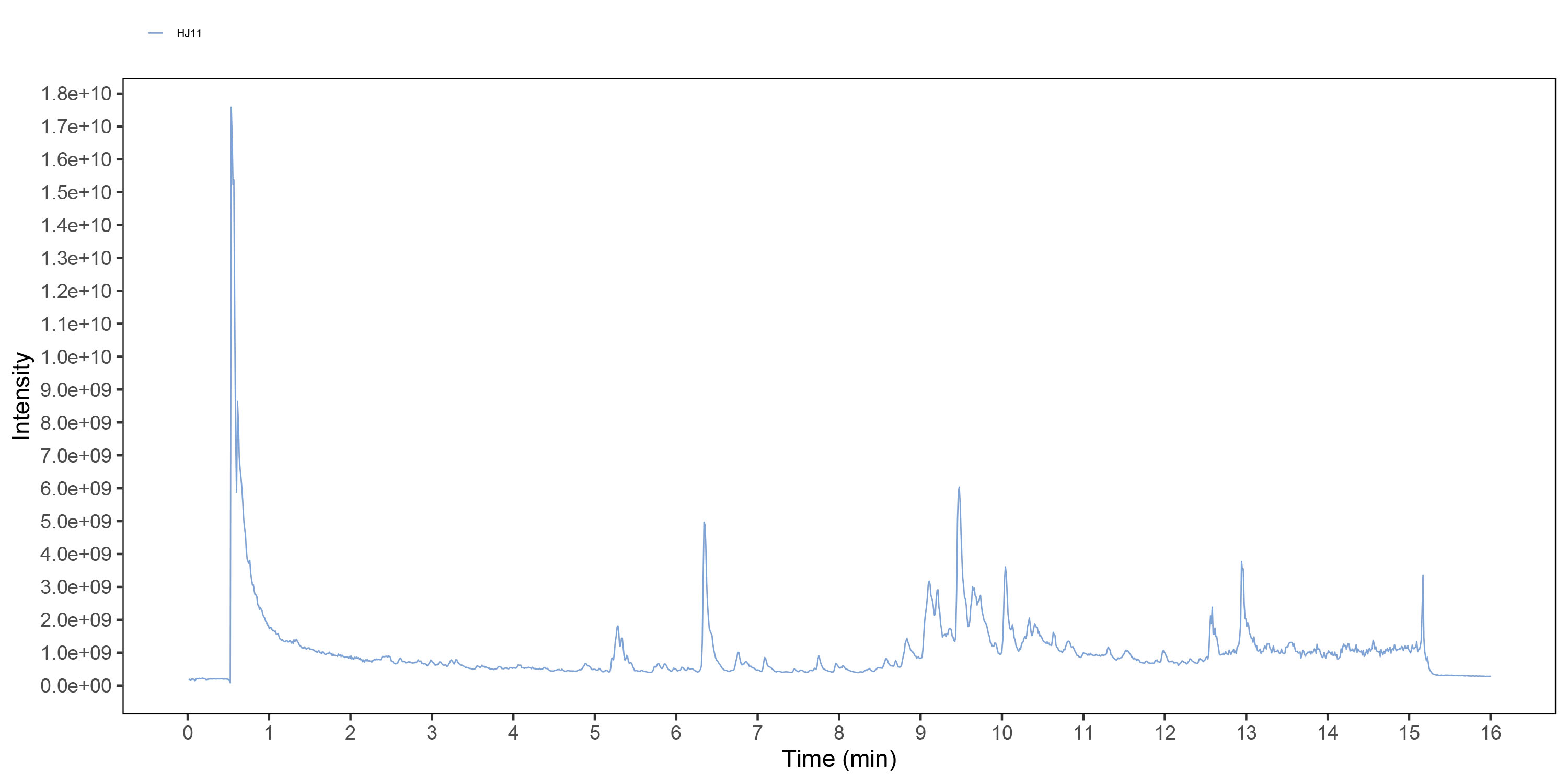

Supplement: Supplementary file 1 [file foods-13-01586-s001.zip › supplement S1/negative ion/NEG-T-13.jpg]

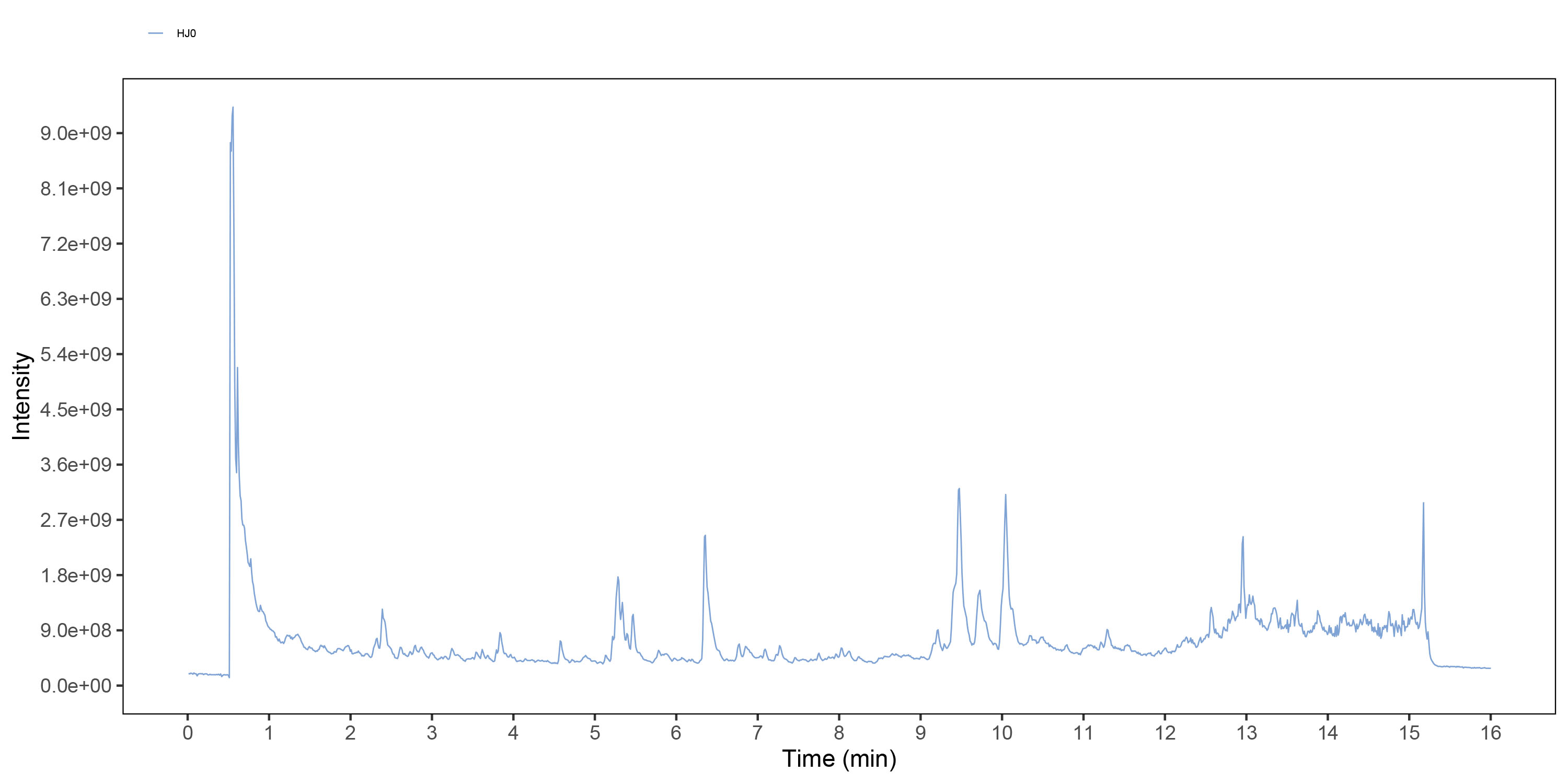

Supplement: Supplementary file 1 [file foods-13-01586-s001.zip › supplement S1/negative ion/NEG-T-2.jpg]

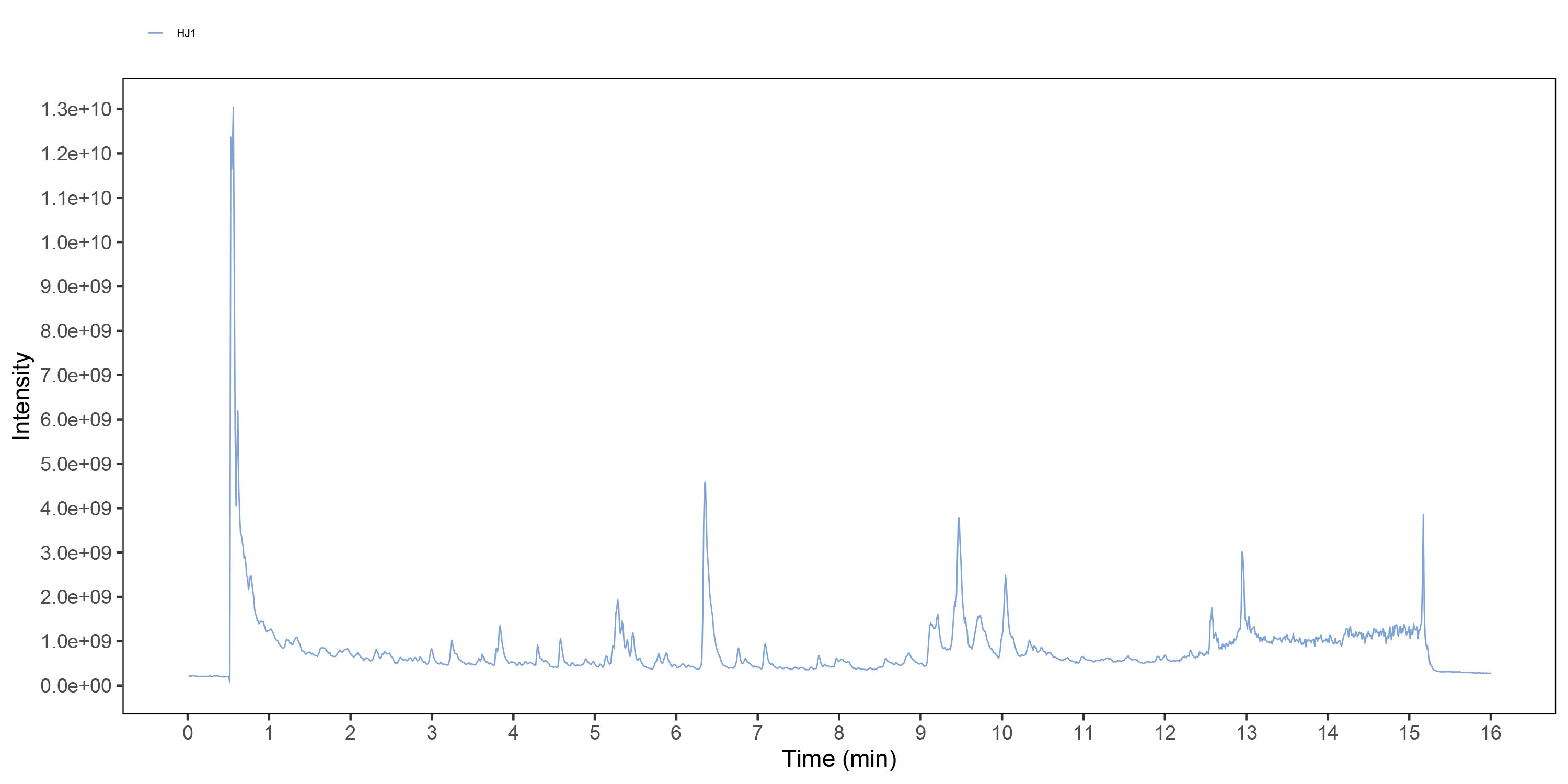

Supplement: Supplementary file 1 [file foods-13-01586-s001.zip › supplement S1/negative ion/NEG-T-3.jpg]

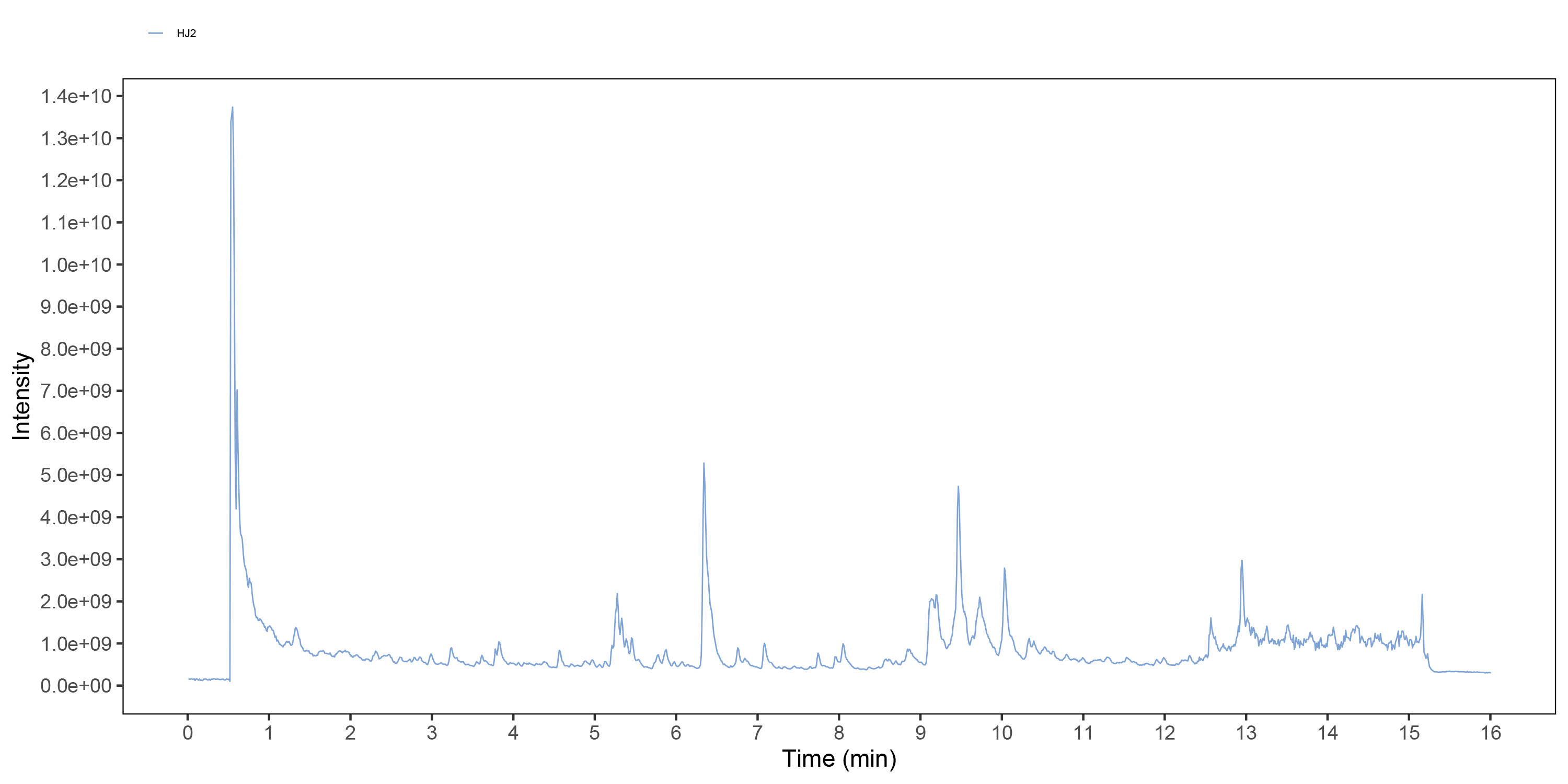

Supplement: Supplementary file 1 [file foods-13-01586-s001.zip › supplement S1/negative ion/NEG-T-4.jpg]

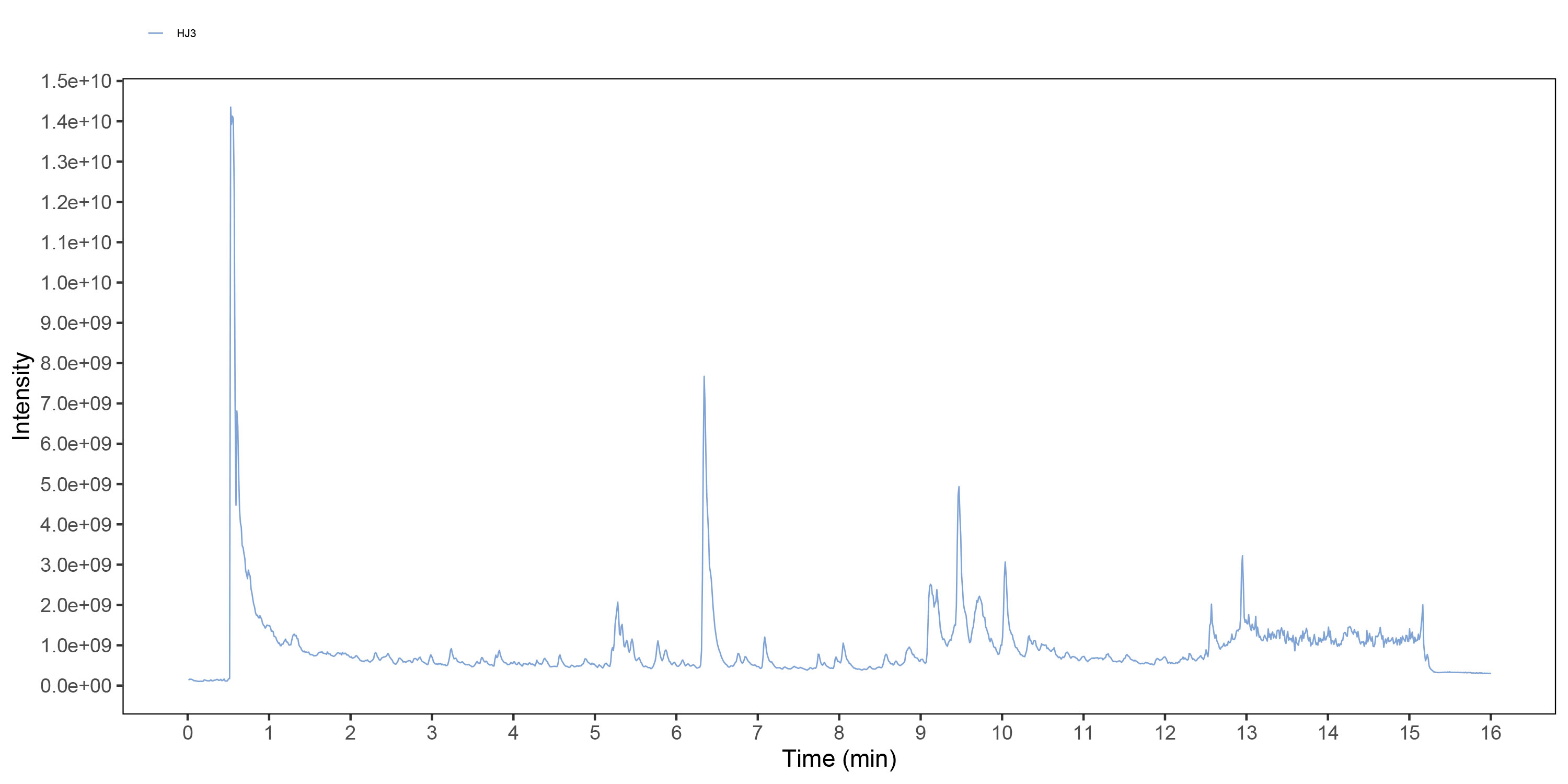

Supplement: Supplementary file 1 [file foods-13-01586-s001.zip › supplement S1/negative ion/NEG-T-5.jpg]

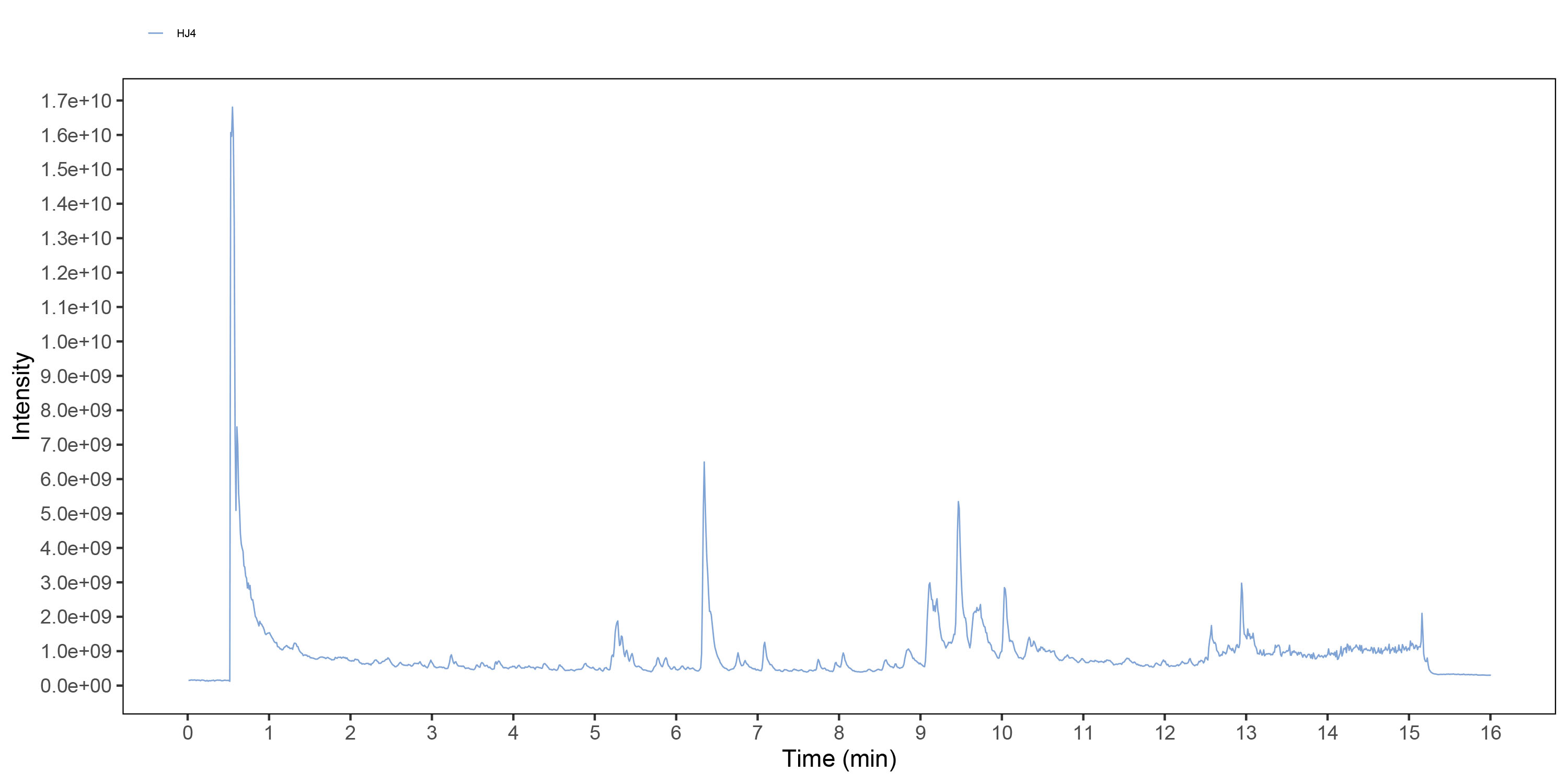

Supplement: Supplementary file 1 [file foods-13-01586-s001.zip › supplement S1/negative ion/NEG-T-6.jpg]

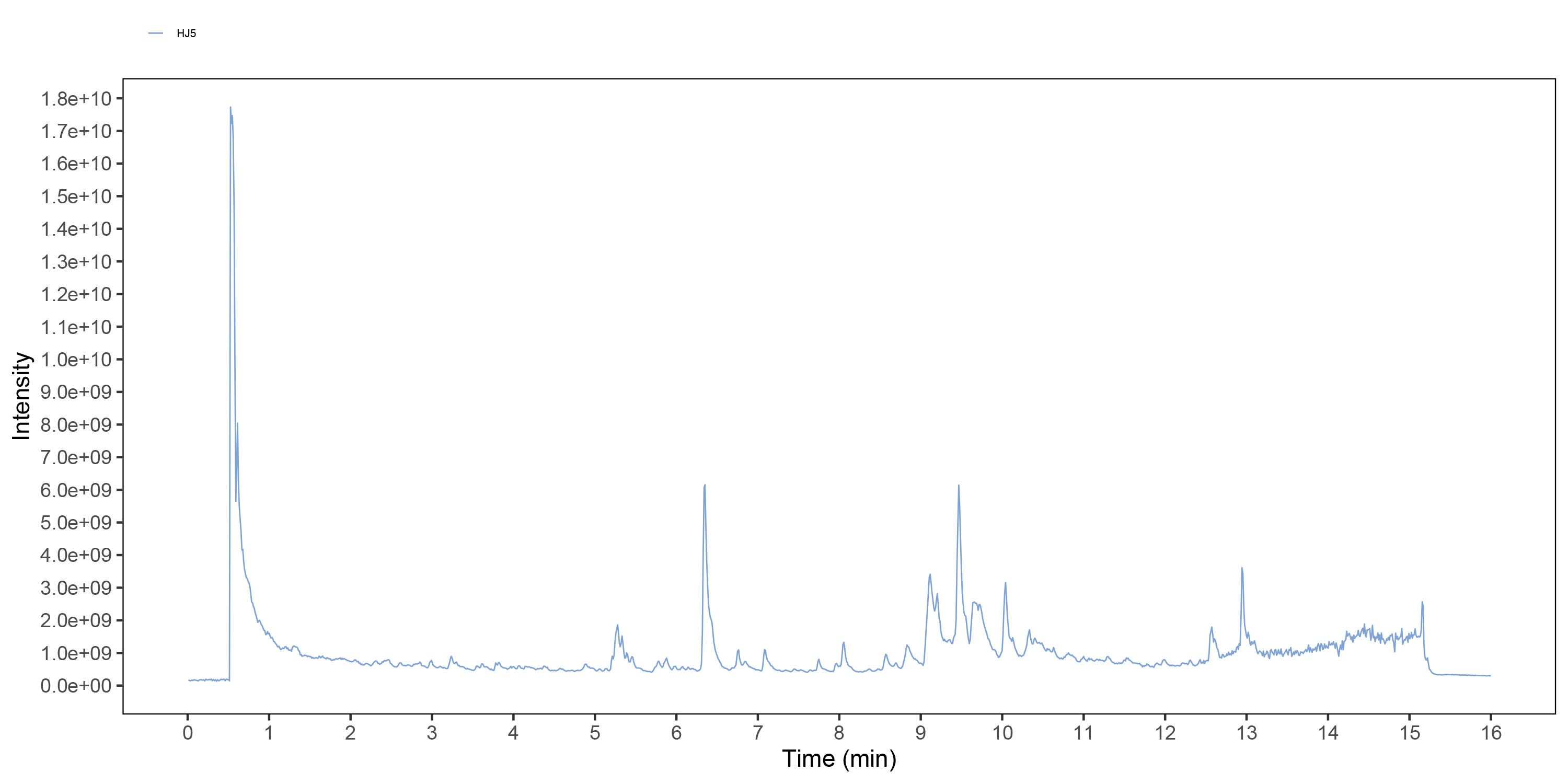

Supplement: Supplementary file 1 [file foods-13-01586-s001.zip › supplement S1/negative ion/NEG-T-7.jpg]

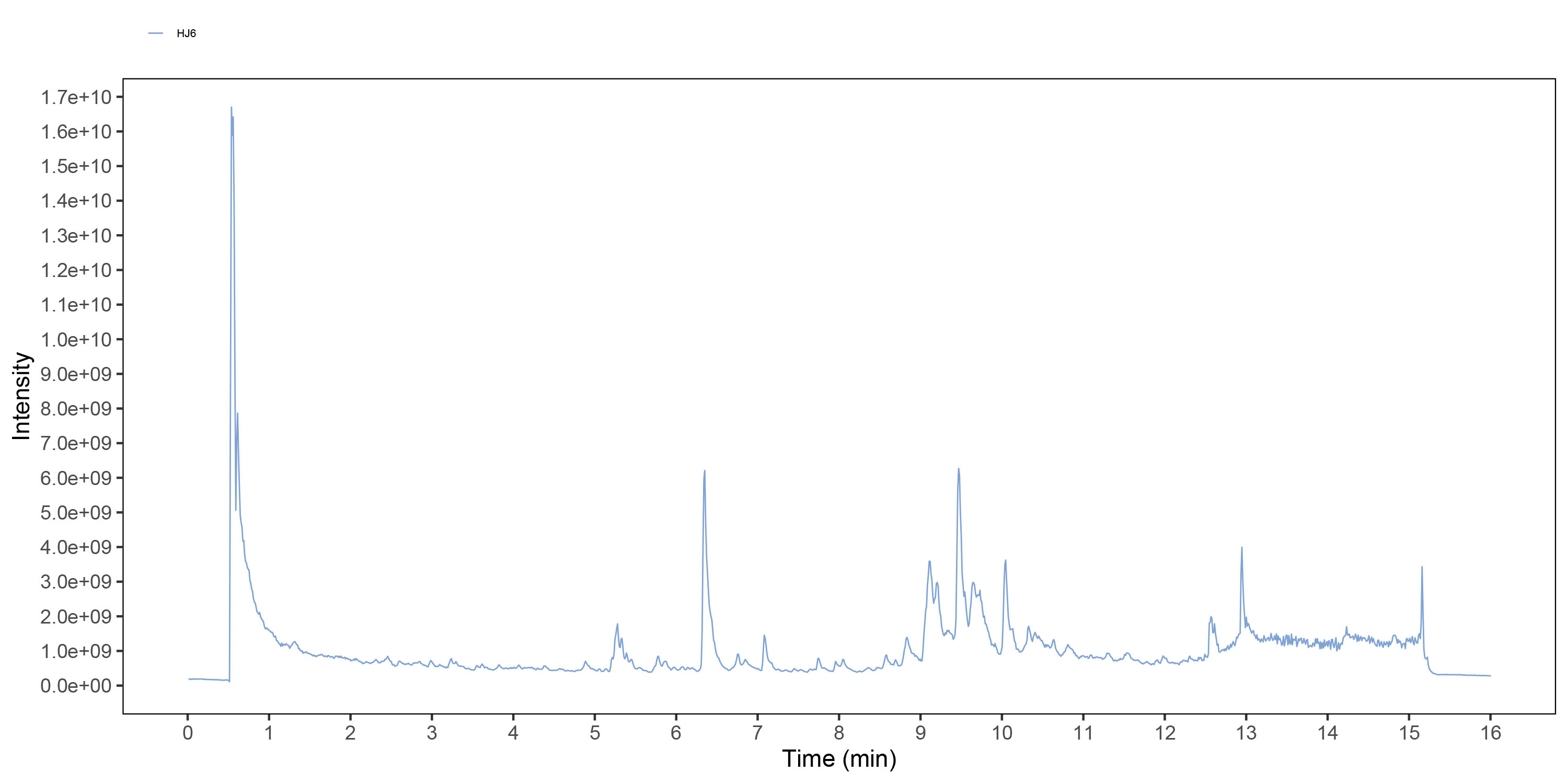

Supplement: Supplementary file 1 [file foods-13-01586-s001.zip › supplement S1/negative ion/NEG-T-8.jpg]

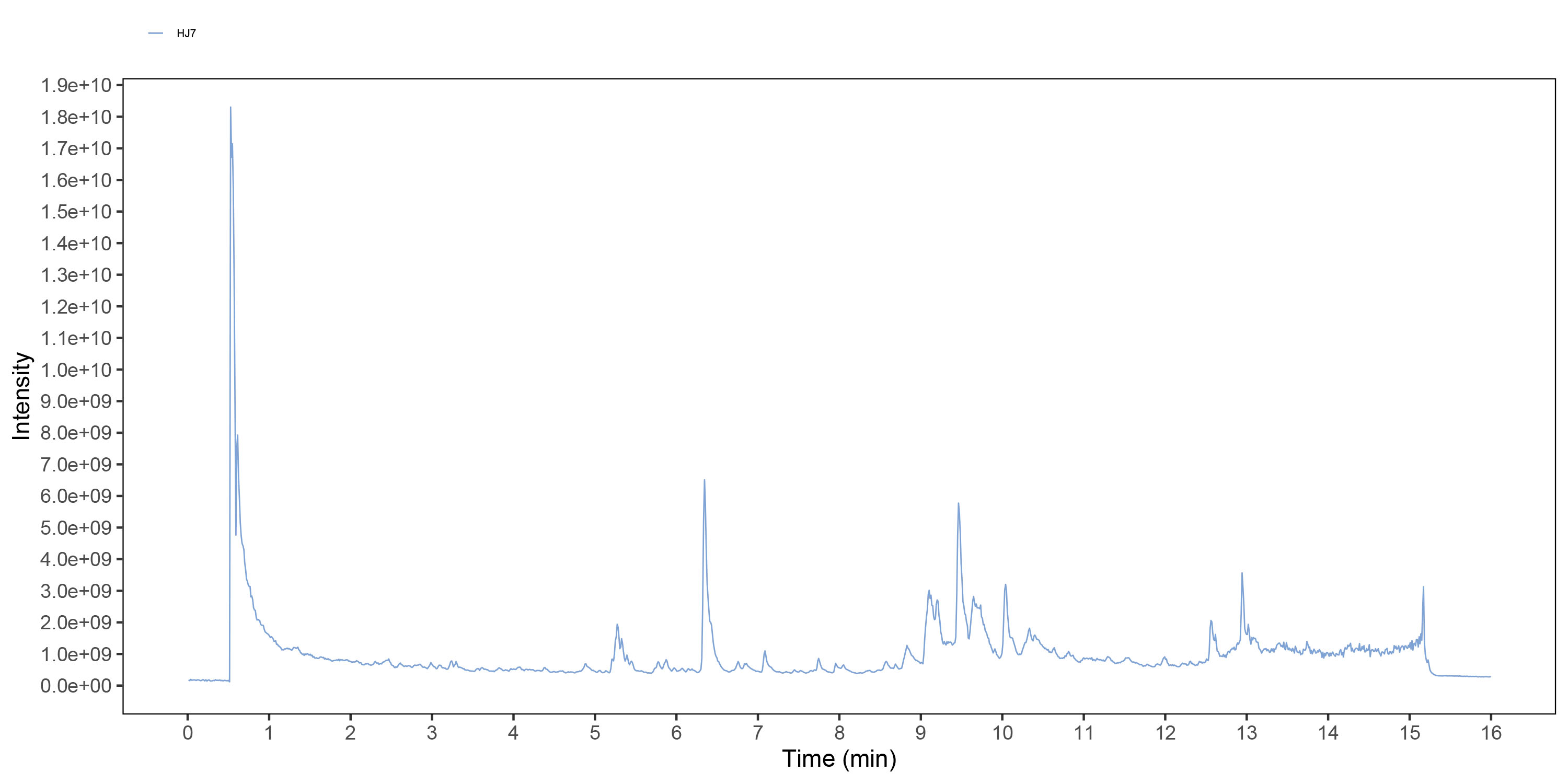

Supplement: Supplementary file 1 [file foods-13-01586-s001.zip › supplement S1/negative ion/NEG-T-9.jpg]

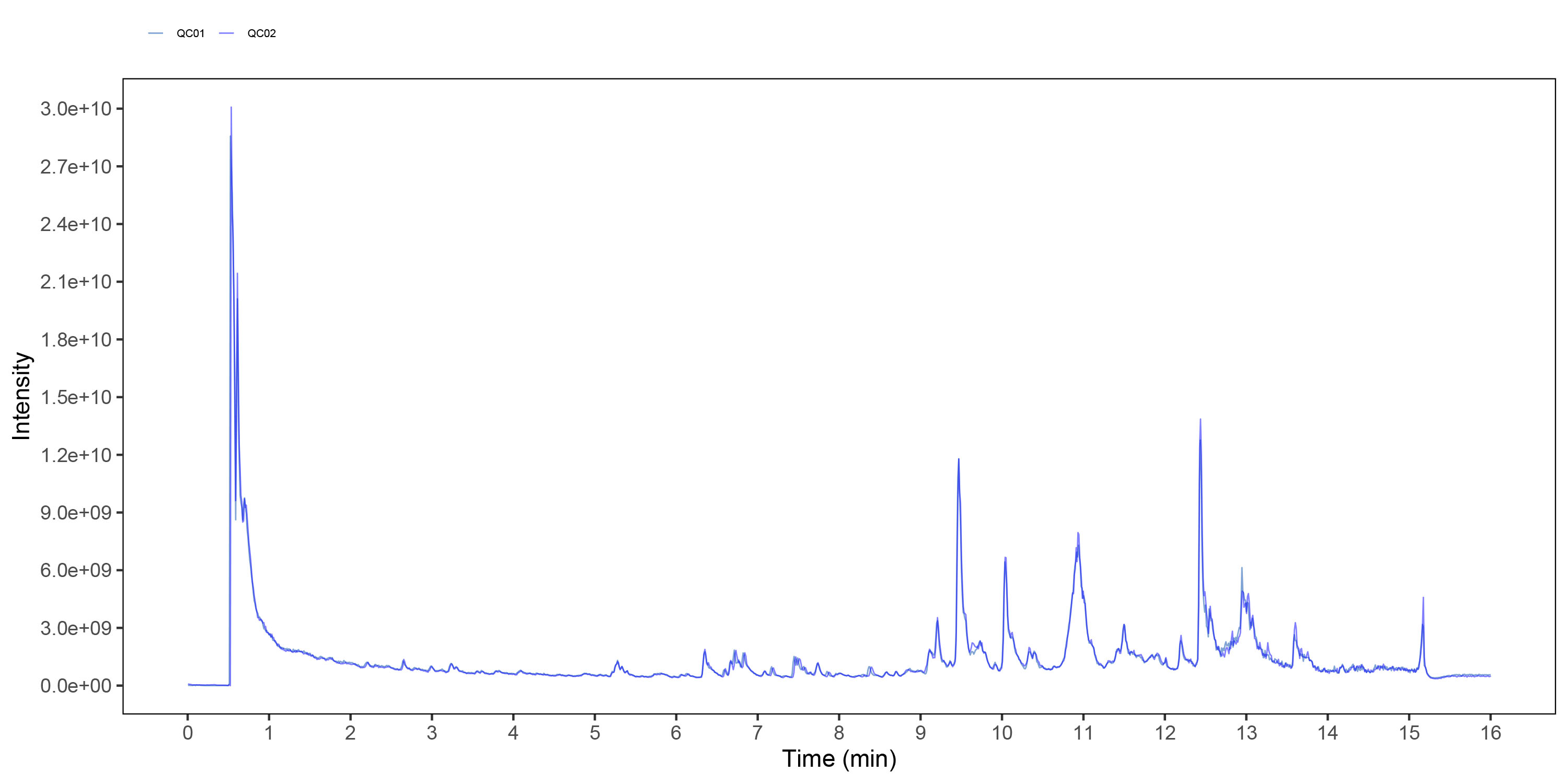

Supplement: Supplementary file 1 [file foods-13-01586-s001.zip › supplement S1/positive ion/POS-T-1.jpg]

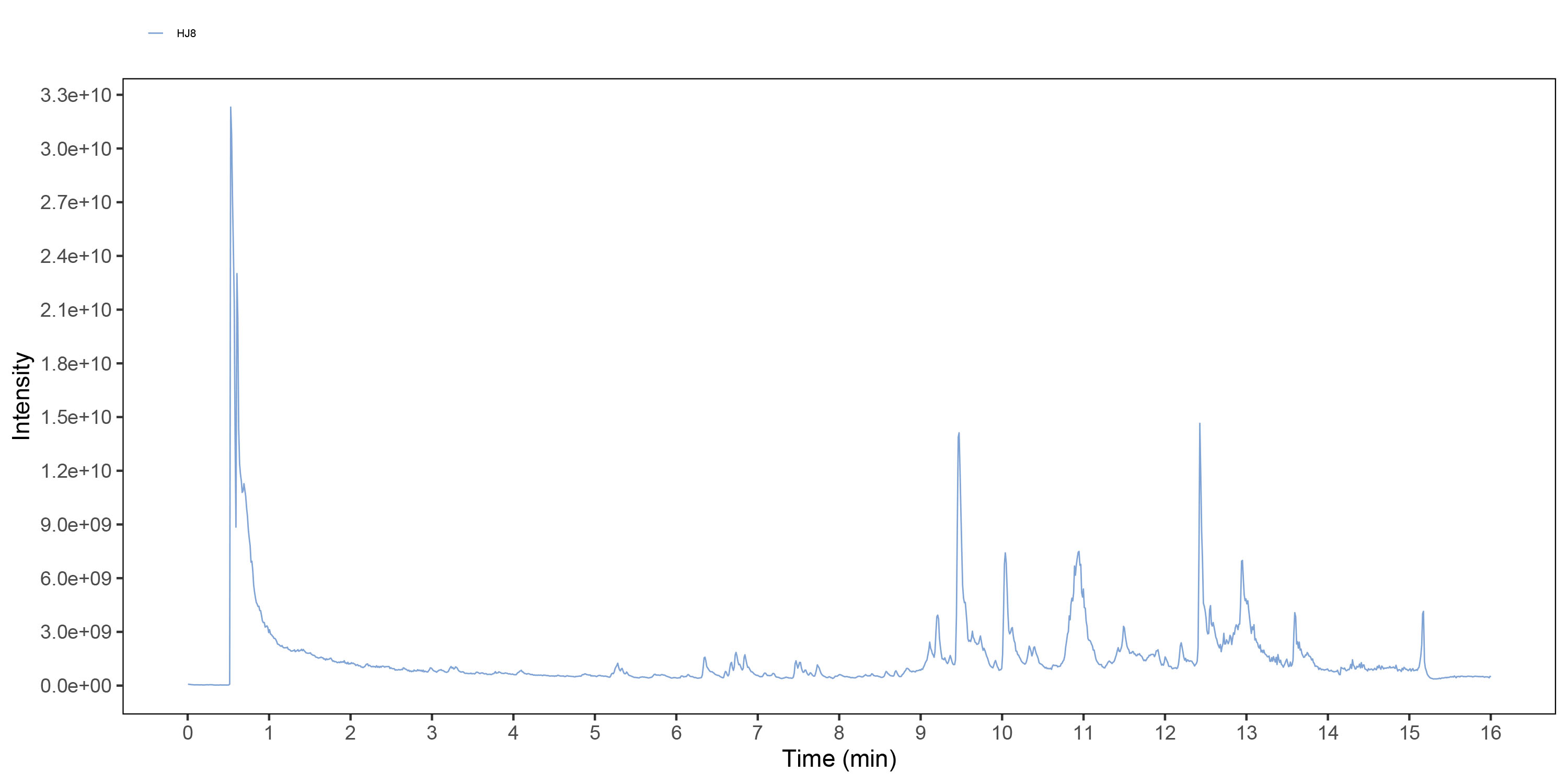

Supplement: Supplementary file 1 [file foods-13-01586-s001.zip › supplement S1/positive ion/POS-T-10.jpg]

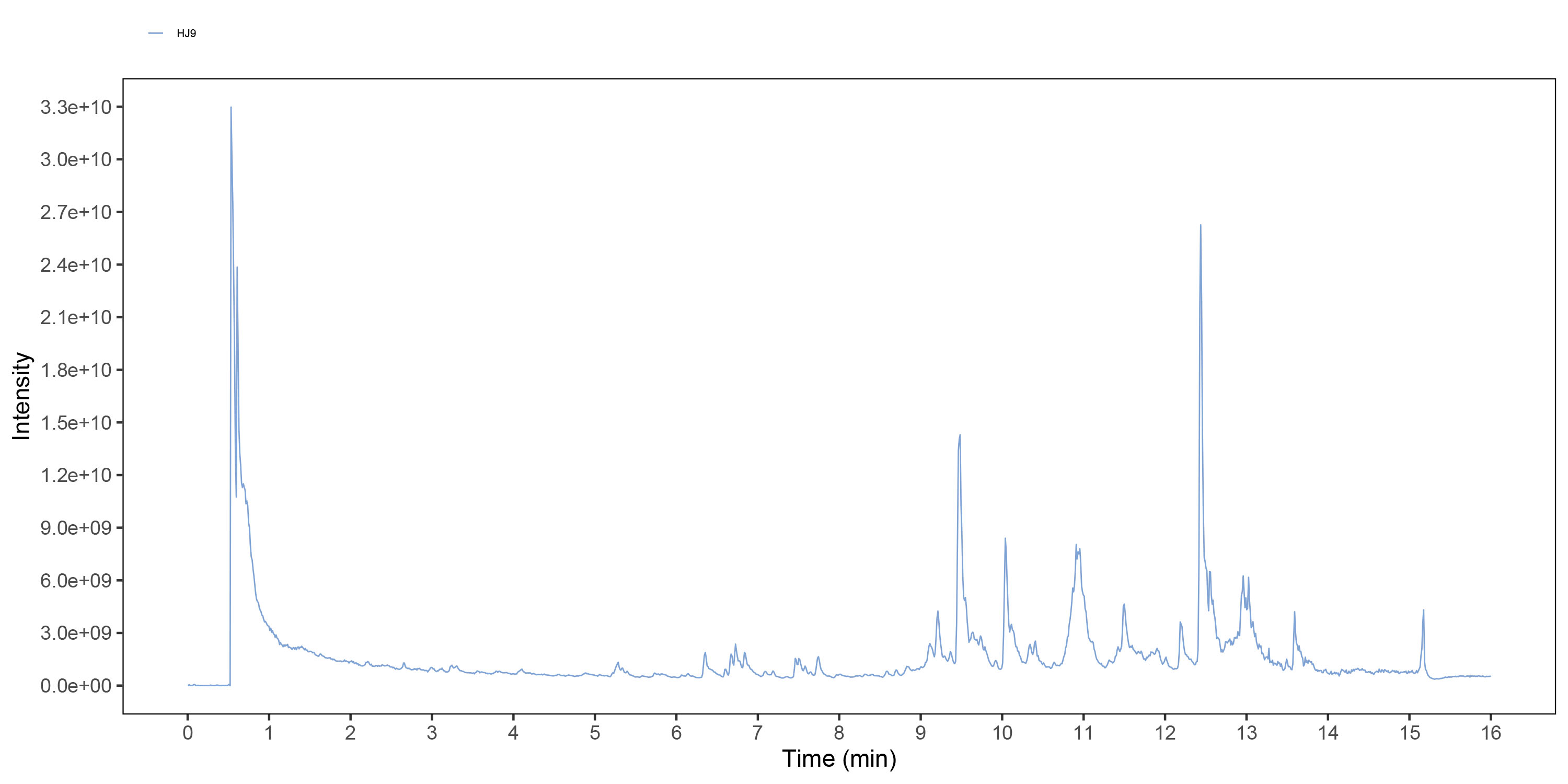

Supplement: Supplementary file 1 [file foods-13-01586-s001.zip › supplement S1/positive ion/POS-T-11.jpg]

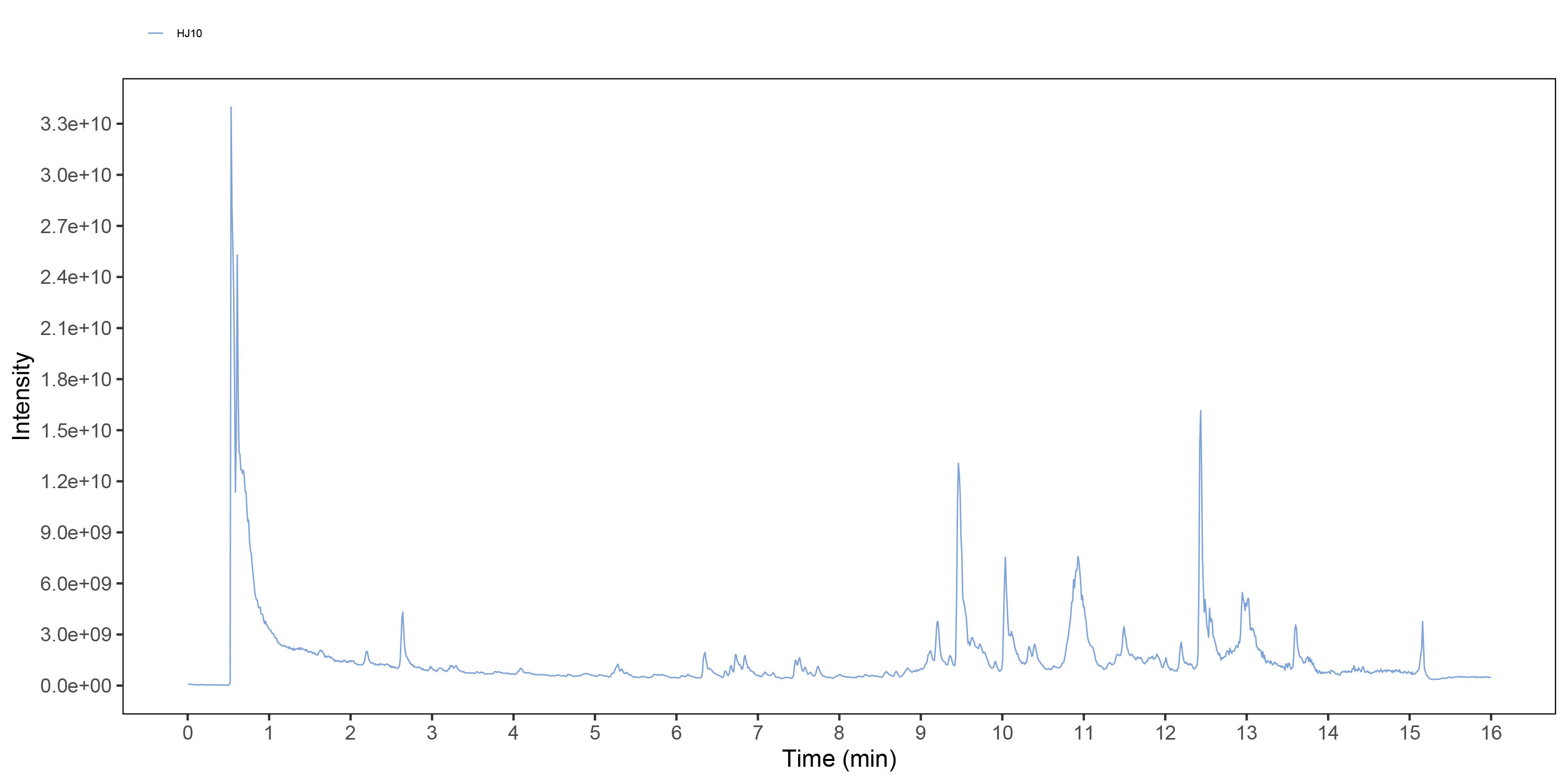

Supplement: Supplementary file 1 [file foods-13-01586-s001.zip › supplement S1/positive ion/POS-T-12.jpg]

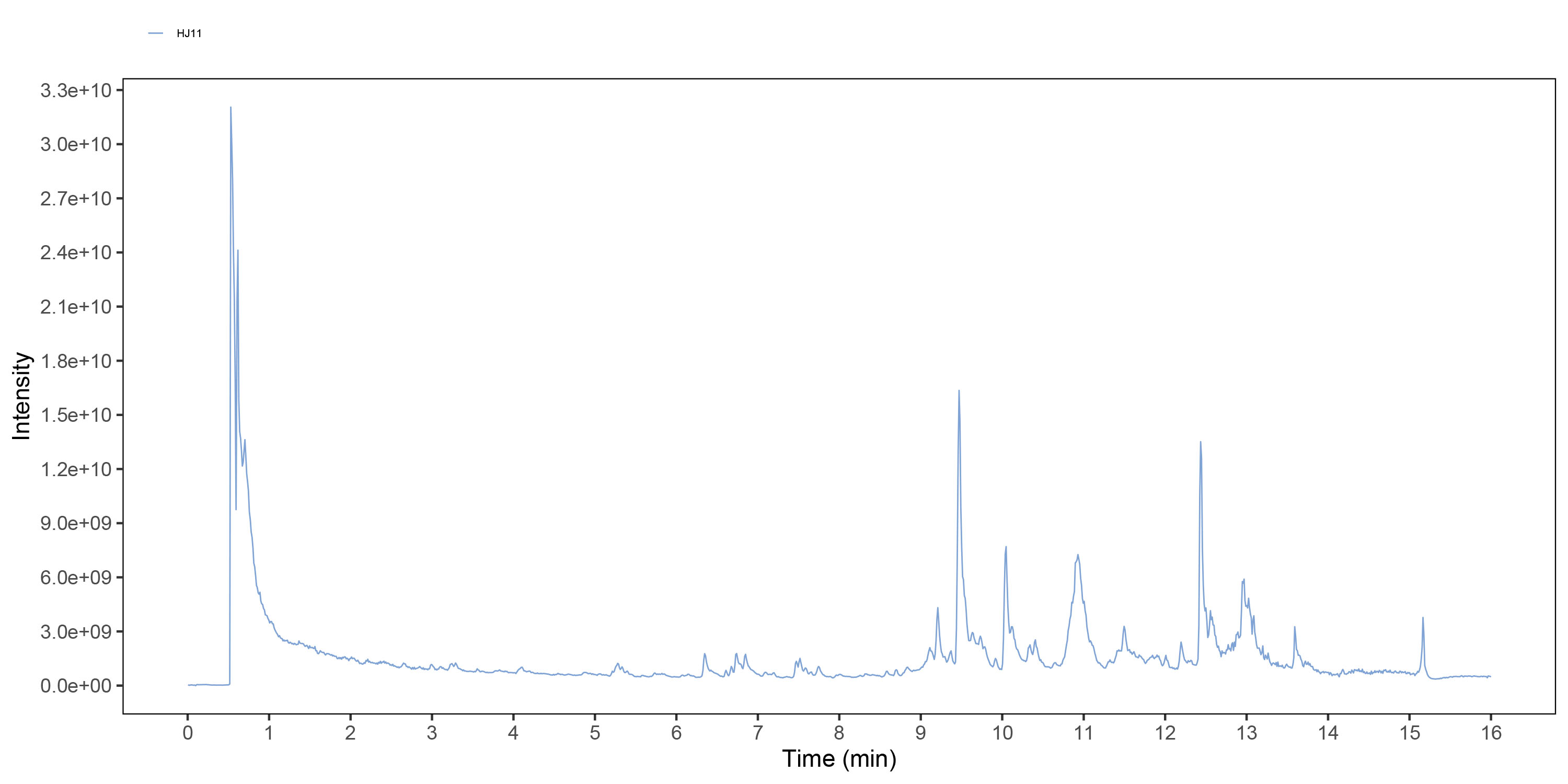

Supplement: Supplementary file 1 [file foods-13-01586-s001.zip › supplement S1/positive ion/POS-T-13.jpg]

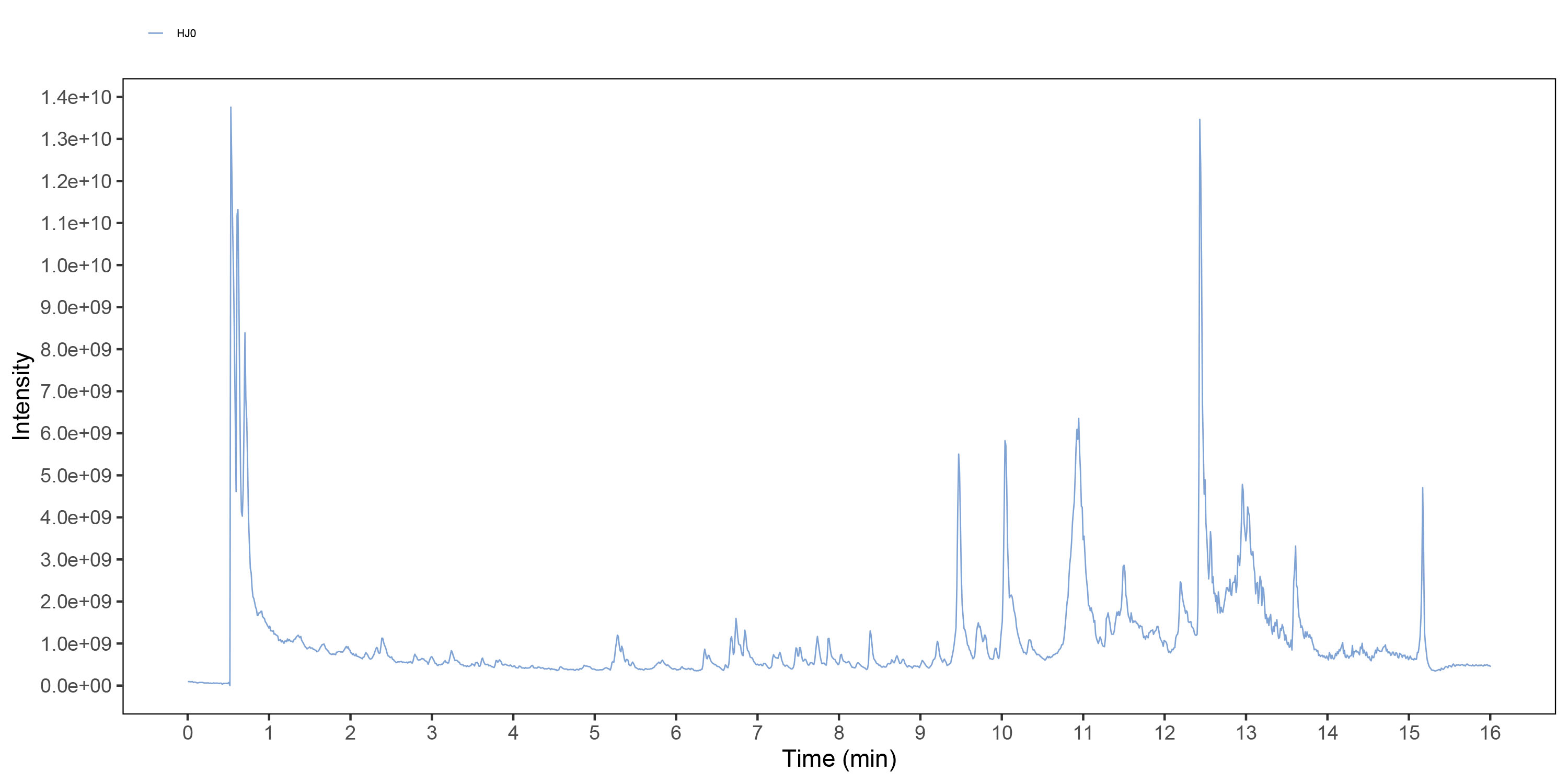

Supplement: Supplementary file 1 [file foods-13-01586-s001.zip › supplement S1/positive ion/POS-T-2.jpg]

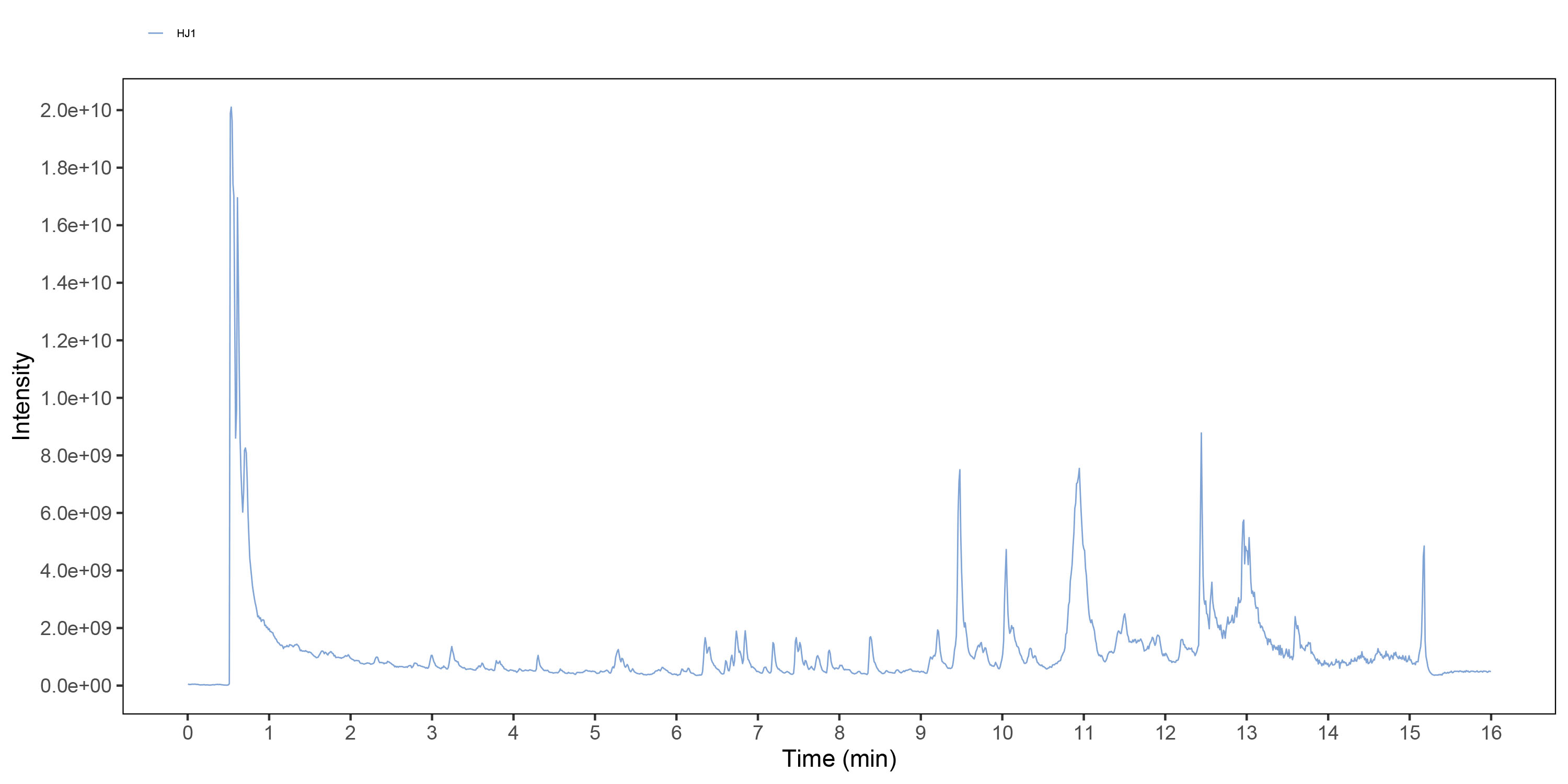

Supplement: Supplementary file 1 [file foods-13-01586-s001.zip › supplement S1/positive ion/POS-T-3.jpg]

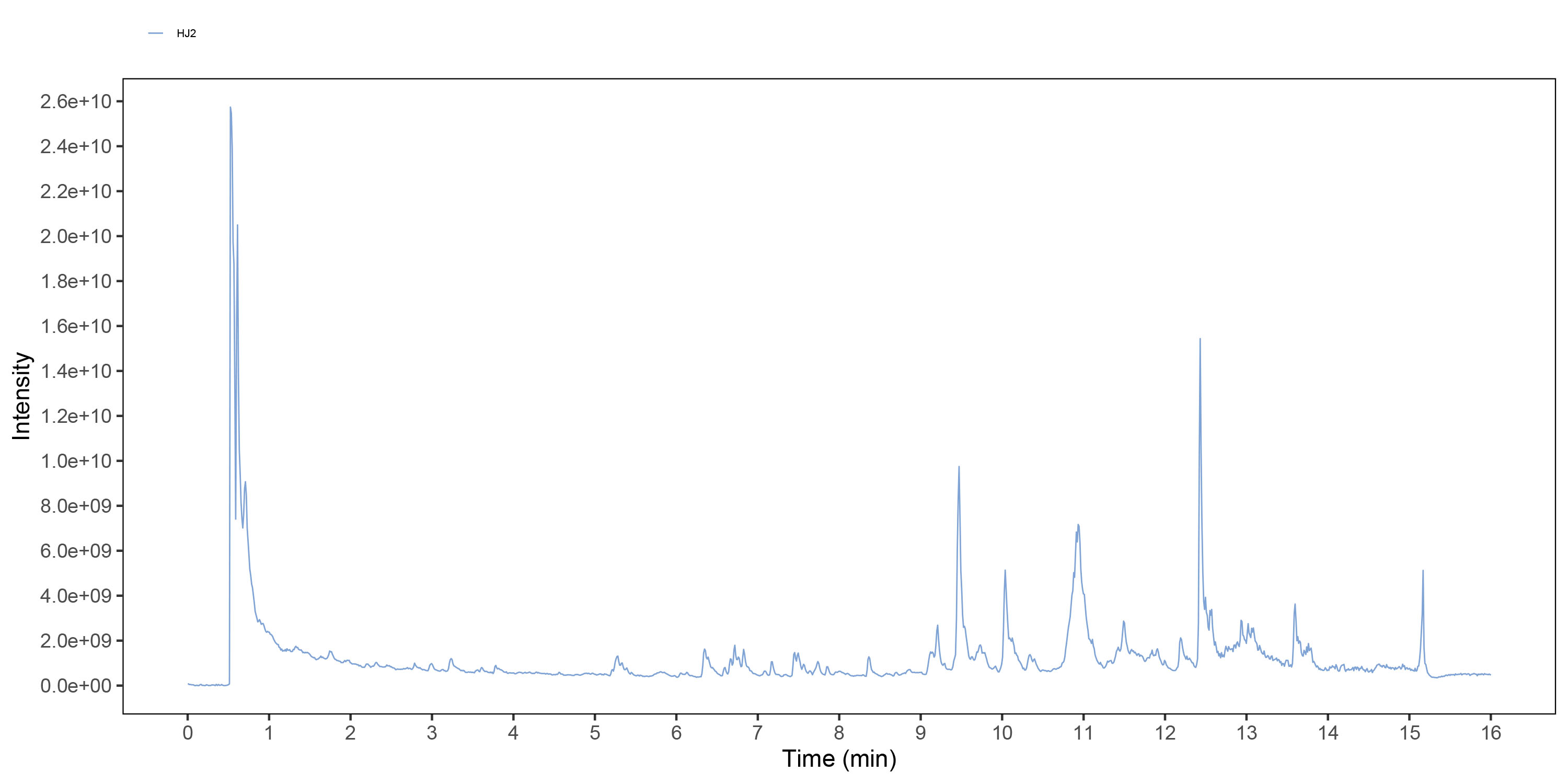

Supplement: Supplementary file 1 [file foods-13-01586-s001.zip › supplement S1/positive ion/POS-T-4.jpg]

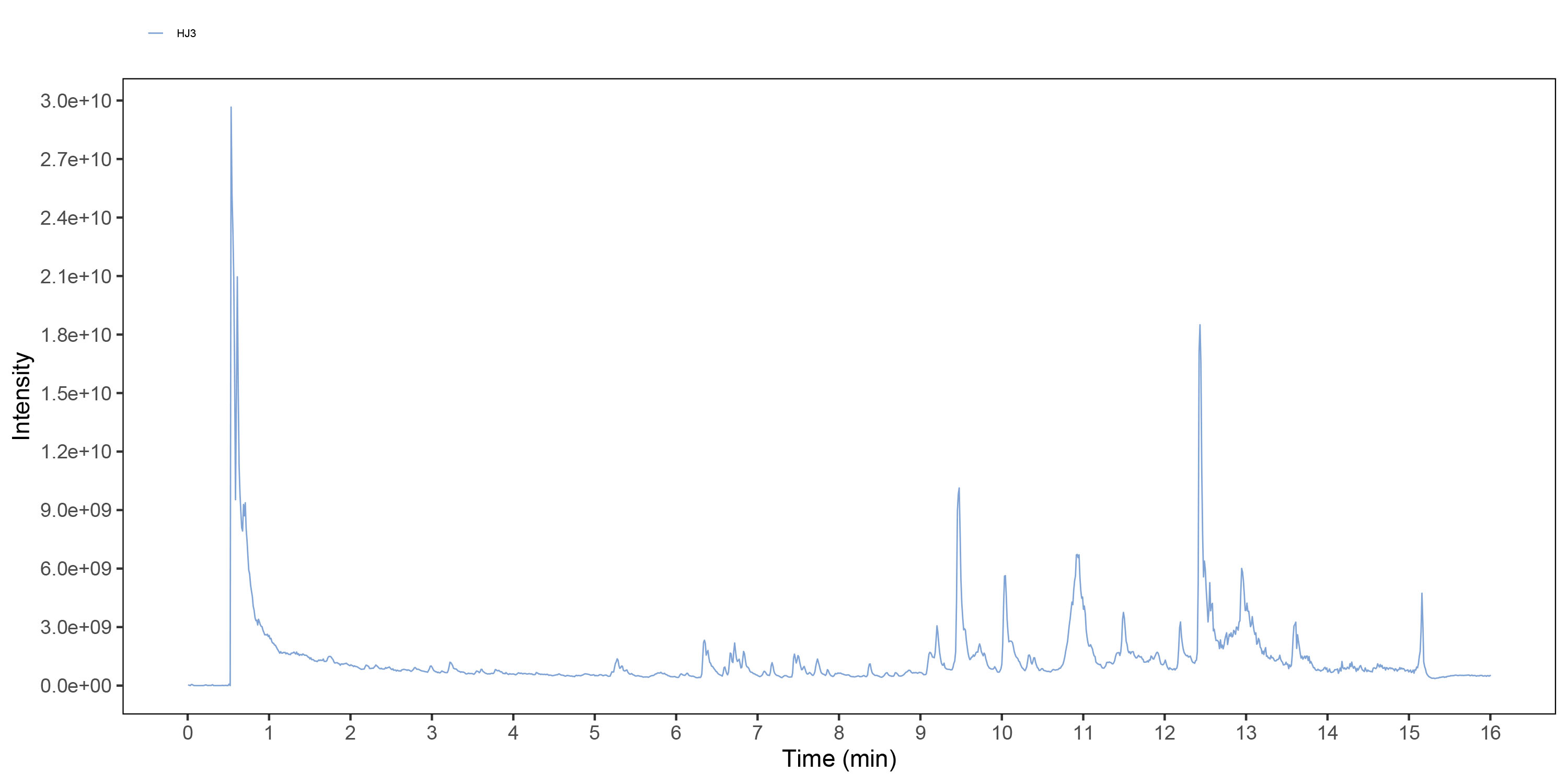

Supplement: Supplementary file 1 [file foods-13-01586-s001.zip › supplement S1/positive ion/POS-T-5.jpg]

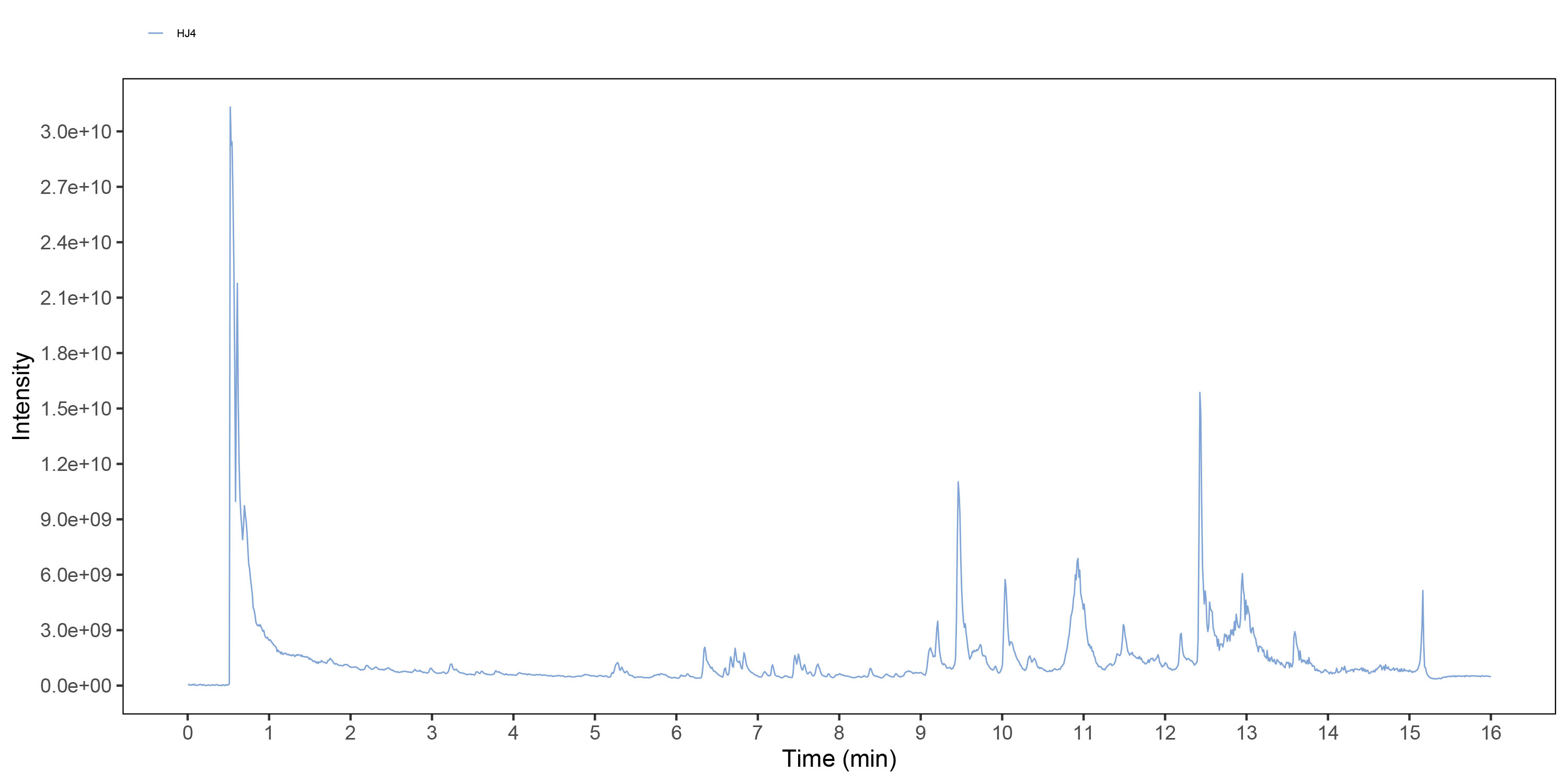

Supplement: Supplementary file 1 [file foods-13-01586-s001.zip › supplement S1/positive ion/POS-T-6.jpg]

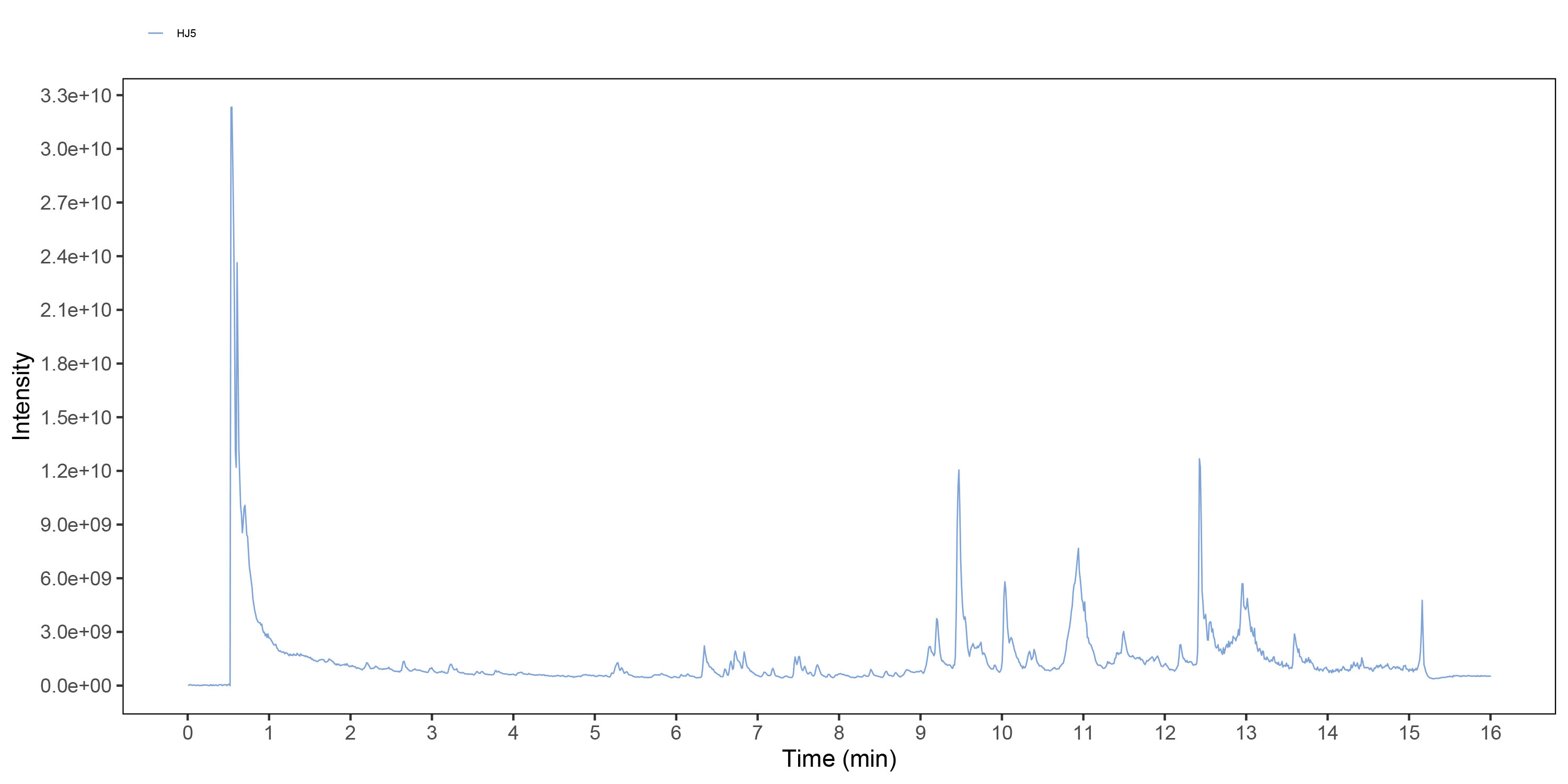

Supplement: Supplementary file 1 [file foods-13-01586-s001.zip › supplement S1/positive ion/POS-T-7.jpg]

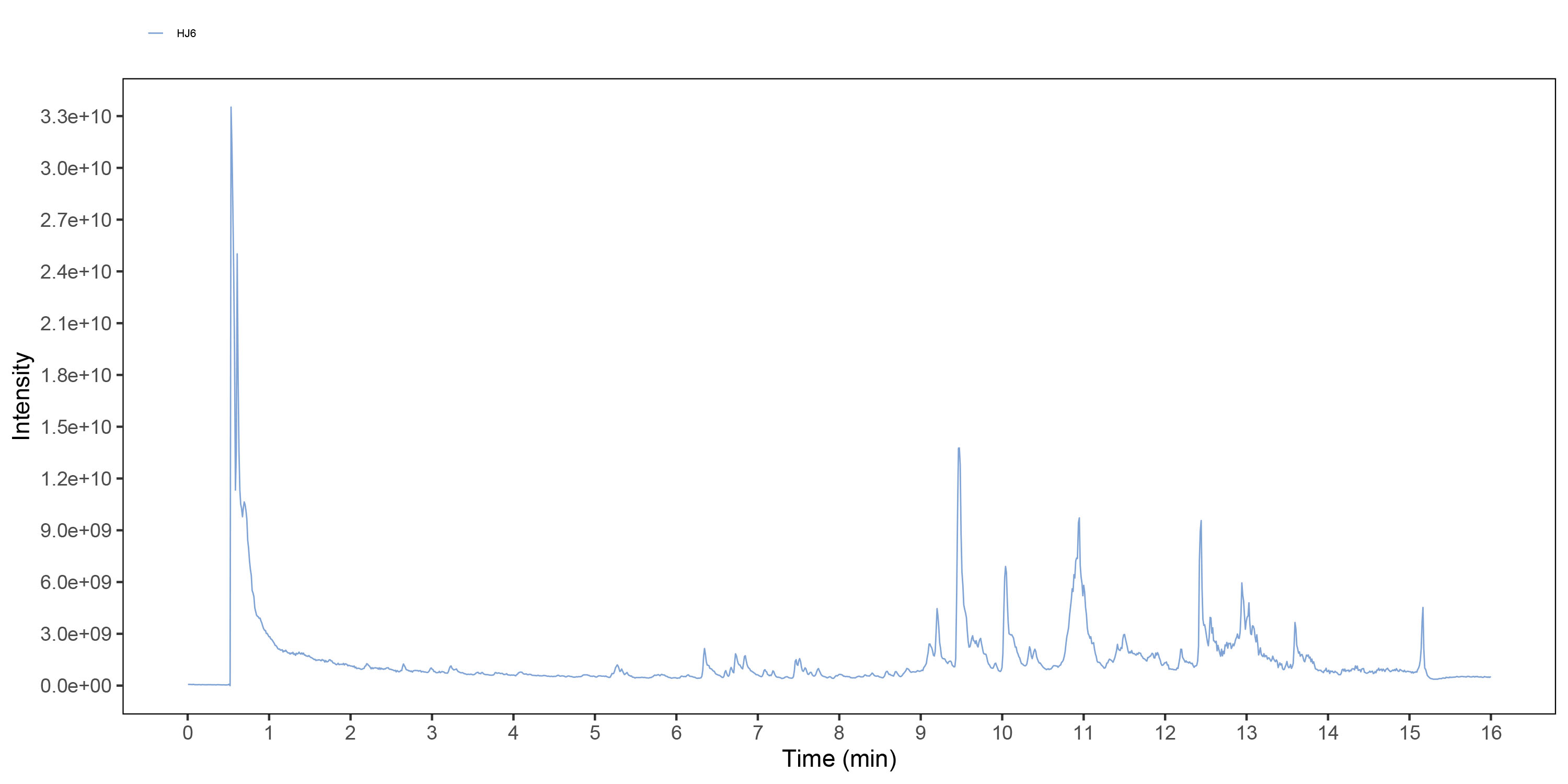

Supplement: Supplementary file 1 [file foods-13-01586-s001.zip › supplement S1/positive ion/POS-T-8.jpg]

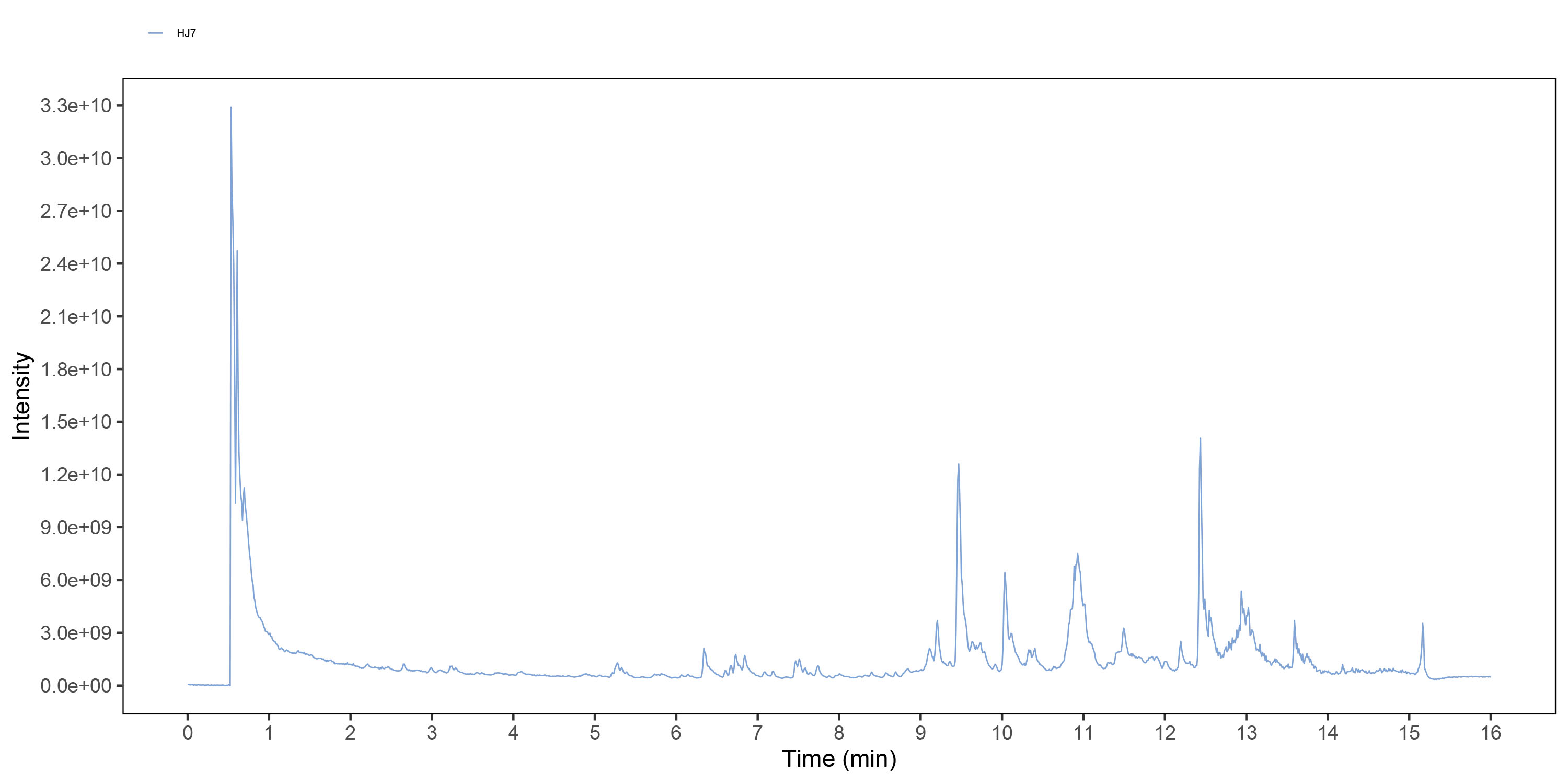

Supplement: Supplementary file 1 [file foods-13-01586-s001.zip › supplement S1/positive ion/POS-T-9.jpg]
